# Describing mortality trends for major cancer sites in 133 intermediate regions of Brazil and an ecological study of its causes

**DOI:** 10.1186/s12885-019-6184-1

**Published:** 2019-10-11

**Authors:** Alessandro Bigoni, José Leopoldo Ferreira Antunes, Elisabete Weiderpass, Kristina Kjærheim

**Affiliations:** 1 Department of Epidemiology, School of Public Health, University of São Paulo, Av. Dr. Arnaldo 715, Pacaembu, Sao Paulo, SP CEP: 01246-904 Brazil; 2 International Agency for Research on Cancer (IARC), WHO, Lyon, France; 3 Cancer Registry of Norway, Oslo, Norway

**Keywords:** Cancer, Mortality, Health services, Time-series, Brazil

## Abstract

**Background:**

In Brazil, 211 thousand (16.14%) of all death certificates in 2016 identified cancer as the underlying cause of death, and it is expected that around 320 thousand will receive a cancer diagnosis in 2019. We aimed to describe trends of cancer mortality from 1996 to 2016, in 133 intermediate regions of Brazil, and to discuss macro-regional differences of trends by human development and healthcare provision.

**Methods:**

This ecological study assessed georeferenced official data on population and mortality, health spending, and healthcare provision from Brazilian governmental agencies. The regional office of the United Nations Development Program provided data on the Human Development Index in Brazil. Deaths by misclassified or unspecified causes (garbage codes) were redistributed proportionally to known causes. Age-standardized mortality rates used the world population as reference. Prais-Winsten autoregression allowed calculating trends for each region, sex and cancer type.

**Results:**

Trends were predominantly on the increase in the North and Northeast, whereas they were mainly decreasing or stationary in the South, Southeast, and Center-West. Also, the variation of trends within intermediate regions was more pronounced in the North and Northeast. Intermediate regions with higher human development, government health spending, and hospital beds had more favorable trends for all cancers and many specific cancer types.

**Conclusions:**

Patterns of cancer trends in the country reflect differences in human development and the provision of health resources across the regions. Increasing trends of cancer mortality in low-income Brazilian regions can overburden their already fragile health infrastructure. Improving the healthcare provision and reducing socioeconomic disparities can prevent increasing trends of mortality by all cancers and specific cancer types in Brazilian more impoverished regions.

## Background

In Brazil, 211 thousand (16.14%) of all death certificates in 2016 identified cancer as the underlying cause of death, and it is expected that around 320 thousand will receive a cancer diagnosis in 2019, excluding non-melanoma skin cancers [1]. Incidence rates for several types of cancer are increasing over time in Brazil [2], such as breast [3], colon and rectum [4], pancreas [5], prostate [6], some head and neck cancers [7, 8], and lung cancer in women [9]. Cancer incidence trends, however, vary significantly according to region and sex.

Cancer mortality rates are a useful tool to assess the burden of the disease, especially in the absence of population-based cancer registries. The comparison of time trends among different regions in Brazil may provide valuable information to the planning of health strategies, programs, and policies. Most of the scientific literature on mortality in the different regions of Brazil focuses on specific types of cancer. Although the assessment of mortality trends gives depth to the understanding of the epidemiologic behavior of particular cancer types, it does not add to the discussion about socioeconomic risk factors and access to health care services, which are relevant to the planning of cancer management and control.

The assessment of mortality trends usually refers to the state or the country level. This approach does not take into consideration socioeconomic disparities and inequalities in the access to health care services within such macro-regional geographical units and may thus fail to inform on a potentially considerable variation in cancer mortality [10, 11]. On the other hand, many ecological studies about cancer outcomes and determinants assess data at the small area level, which are more homogeneous from the socioeconomic standpoint. However, studies in small areas do not take into account that the population usually demands health services located outside their residential inner circle.

Brazil namely implemented universal access to health services in 1988. The Unified Health System (*Sistema Único de Saúde*; SUS) aims to provide free-of-charge treatment, preventive actions, and programs for health promotion throughout the country. However, Brazil is affected by severe socioeconomic disparities, and its health system has suffered from chronic underfunding and reduced access in poorer regions. The SUS is supplemented by the private sector, which provides out-of-pocket services and health insurance, according to its users’ ability to pay. Although the proportion of private health insurance has increased, almost 75% of the population still relies solely on the SUS. It is estimated that more than 85% of the country has access to primary care via the Family Health Program, a strategy implemented by the SUS to expand access, including to rural areas. Inequalities in access to health services is still a major issue in the country, and specialized medical care is mostly centralized in the main metropolises in the South and Southeast regions [12]. The lack of health care infrastructure in some Brazilian regions, especially the North and Northeast, makes it necessary for the inhabitants of inland municipalities to resort to the nearest metropolitan city when affected by complex diseases such as cancer. This option can be cost prohibitive for an already deprived population, thus influencing mortality rates in the region.

We present here an ecological analysis of cancer mortality time trends by intermediate region level, considering that these geographic units are less heterogeneous than states and macro-regions and that they constitute the reference in demand for health services. We describe here trends of cancer mortality for all cancers combined and eight cancer types from 1996 to 2016, in 133 intermediate regions distributed by 27 states (five macro-regions) of Brazil. Furthermore, we aimed to discuss the trends in light of the differences in the provision of healthcare, human development and governmental expenditure on health.

## Methods

### Data sources

This ecological assessment used mortality data from 1996 to 2016, obtained within the official system of information on mortality maintained by the Brazilian Ministry of Health. The first year of monitoring was 1996 when the Brazilian Mortality Information System started using the tenth revision of the International Classification of Diseases (ICD-10), which modified the coding of cancer deaths substantially [13].

Information on the Human Development Index (HDI) was obtained in the Atlas of Human Development, prepared by the Brazilian section of the United Nations Development Program, with data related to 2010. HDI is a composite index assembling information on life expectancy, education, and per capita income. Governmental agencies (the National Registry of Health Facilities and the Information System on Public Health Budgets) informed data on hospital beds, per 1000 inhabitants (a marker of the overall provision of healthcare), and per capita government spending on health in each intermediate region. Health spending was measured in Brazilian Reals, the official currency in the country. Data for the number of beds and government spending refer to 2016. The currency exchange rate is variable; in the middle of 2016, one US dollar was equivalent to 3.20 Brazilian Reals. These indicators were categorized by quartile in order to assess correlations with cancer mortality trends by using Pearson’s correlation and p for trend.

The Brazilian Institute of Geography and Statistics provided demographic data (the number of inhabitants in each municipality, as distributed by sex and age group) relative to censuses performed in 2000 and 2010 and intercensal estimates for the remaining years. The georeference of deaths in intermediate regions considered the municipality of residence filled in the death certificate.

The distribution of deaths was assessed at the intermediate area level, as demarcated by the latest official division of Brazilian regions [14]. This newly-defined system provides a regional division in which the units in each area have meaningful interactions within themselves, taking into consideration business connections and the routes of communication among people and municipality in each region. The definition of intermediate regions also considered that people living in smaller municipality usually demand health services of larger neighboring cities.

### Statistical analysis

Age-standardized mortality rates (ASMRs) were calculated for all types of malignant neoplasms (ICD-10 C00-C97); tumors affecting the head and neck (C00-C14; C32); colon, rectum and anus (C18-C21); pancreas (C25); lung and trachea (C33-C34); breast (C50); prostate (C61); cervix uteri (C53); and stomach cancer (C16).

The estimation of mortality rates included a variable proportion of deaths classified initially as due to “ill-defined causes” and “garbage codes.”. The proportion of these deaths varied over the years and across regions, being more prevalent in low-income regions, though with an overall reduction over time [15]. The Global Burden of Disease (GBD) [16] instructed the method to estimate the exact proportion of deaths by misclassified causes that were attributable to cancer in each year and region. The GBD estimated these proportions based on a review of studies assessing misclassification in death certificates worldwide. We used the GBD proportions to redistribute deaths classified in all garbage codes except for deaths classified in ICD-10 Chapter XVIII: Symptoms, signs and abnormal clinical and laboratory findings, not elsewhere classified” (R00-R99). In these cases, we used the method proposed by França et al. [17], considering that this method was more specific to the Brazilian context. They conducted fieldwork to estimate which proportion of cases attributed to ill-defined and unknown causes of death in Brazil should be redistributed to specified causes, according to the previously known distribution of deaths in each sex, age group, and category of the underlying cause. Both methods described above imply that different proportions of deaths attributed to ill-defined causes and garbage codes should be assigned to specific causes of death in each stratum of age and sex of that specific region. This procedure also takes into consideration that, with the progressive improvement of data quality, the number of deaths with misclassified underlying cause reduced over the years, and a lower proportion were redistributed to our target cancer groups.

The ASMRs accounted for the distribution of age groups (five-year range) in each sex, year, and region. We included deaths with missing information on sex or age by redistributing them proportionally, according to the already known distribution in each region and year. The standardization of age by the direct method used the reference population defined by the World Health Organization [18].

We analyzed mortality from each cancer group by intermediate regions of residence for both sexes and each sex separately. The assessment of trends used Prais-Winsten generalized linear regression, with log-transformed (to base 10) ASMRs as the outcome variable, and year of death as the covariate. This method allows adjusting for the first-order serial autocorrelation, which usually affects timely ordered measurements of social processes. The resulting regression coefficient informs the calculation of the annual percent change (APC) by applying the formula APC = (− 1 + 10^b1^)*100%; and the 95% confidence interval (CI) as (− 1 + 10^b1lower^)*100%; (− 1 + 10^b1upper^)*100%, with “b1lower” and “b1upper” representing the limits of the confidence interval, as described by Antunes and Waldman [19]. The procedure enables classifying the trends as increasing if the resulting APC and its confidence interval are positive, decreasing if they are negative, or stationary if the confidence interval includes the zero [20].

The resulting APCs for each intermediate region was graphically displayed in boxplots, as stratified by macro-regions. Maps depicted georeferenced information on human development, health expenditure, and hospital beds.

The statistical analysis used Stata 15.1 (College Station, Texas, 2018).

## Results

This study encompassed 5570 municipalities aggregated in 133 intermediate regions. From 1996 to 2016, a total of 22,366,860 deaths occurred, of which 3,219,245 had cancer as the underlying cause. During the study period, noticeable differences in trends occurred between intermediate and macro-regions.

**Fig. 1:**
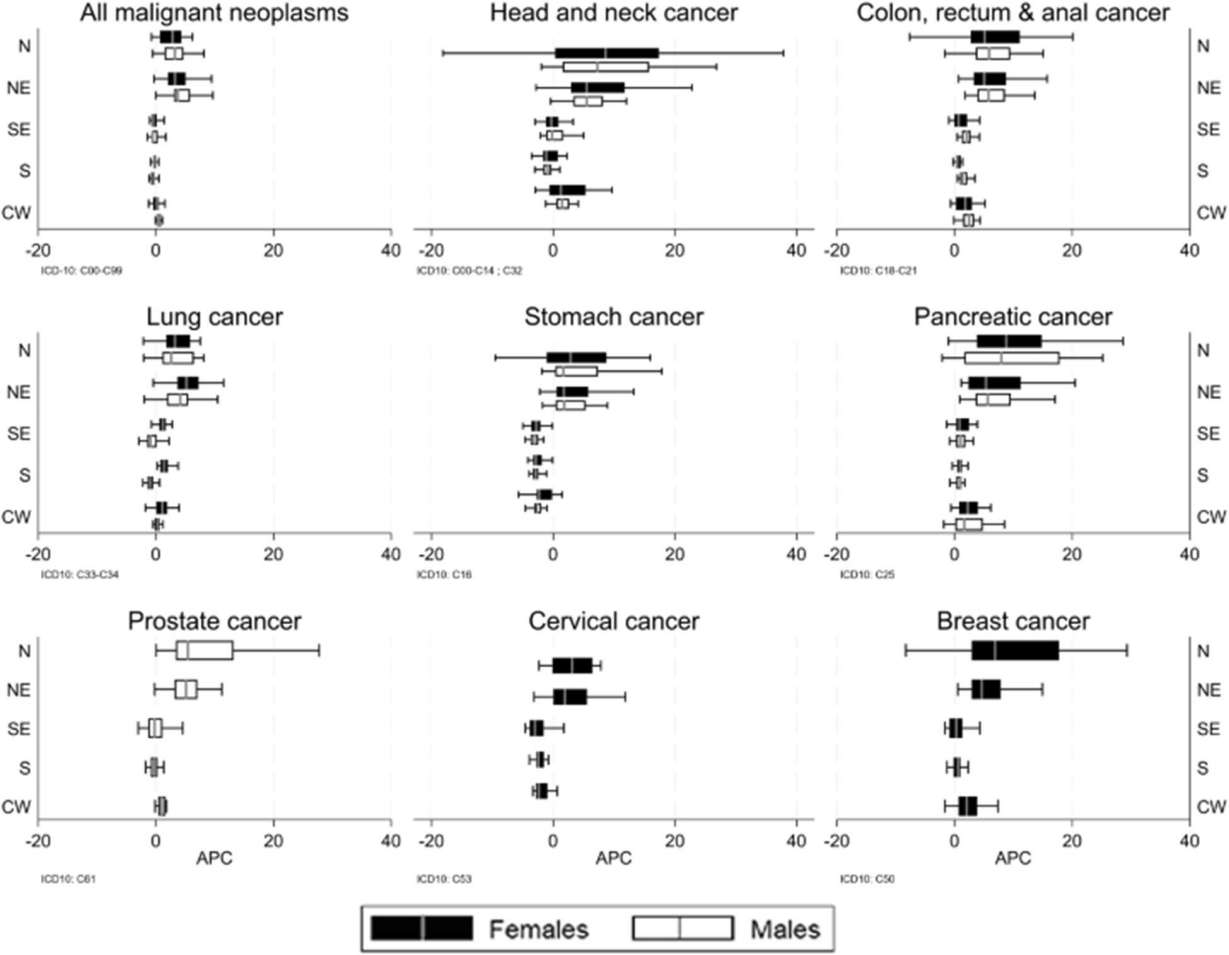
Trends (annual percent change) of cancer mortality in Brazil, 1996–2016. N: North, NE: Northeast, SE: Southeast, S: South, CW: Center-West. Boxplots refer to the variation across intermediate regions, for each macro-region, cancer type, and sex

In the *North region*, overall trends were increasing in all intermediate regions. Median APC values ranged from 1.66% for stomach cancers in males to 8.79% for pancreatic cancer in females (Table 1). The region had the highest variation of trends among all macro-regions, especially for women (Fig. 1). Mortality by lung cancer in women decreased in Porto Velho, in the state of Rondônia (− 2.14% [− 4.20%;-0.03%]), in contrast with increasing trends in all remaining intermediate regions of the country (Additional file 1: Table S1-S5). In the *Northeast region***,** trends were predominantly increasing, with median values for APC ranging from 1.75% for stomach cancer in females to 5.78% for colorectal cancer in males (Table 1). The region also had high median APCs for all types of cancers in both sexes, and the variation of trends was almost as high as in the North region (Fig. 1).

**Table 1:** Trends (annual percent change) of cancer mortality. Median (and interquartile range) APC by sex, macro-region, and type of cancer. Brazil, 1996–2016

		North (n. 22)	Northeast (n. 42)	Southeast (n. 33)	South (n. 21)	Center-West (n. 15)	Brazil (n. 133)
	Sex	Median (IQR)	Median (IQR)	Median (IQR)	Median (IQR)	Median (IQR)	Median (IQR)
All cancers	F	2.81 (0.69; 4.32)	3.23 (2.05; 5.04)	− 0.43 (− 0.74; 0.15)	− 0.25 (− 0.47; − 0.01)	0.12 (− 0.46; 0.46)	0.43 (− 0.37; 2.88)
	M	3.21 (1.47; 4.7)	3.73 (3.1; 5.81)	− 0.58 (− 0.74; 0.28)	− 0.43 (− 0.83; − 0.2)	0.47 (0.22; 0.79)	0.79 (− 0.4; 3.49)
Head & Neck	F	8.6 (0.33; 17.34)	5.52 (2.92; 11.72)	− 0.31 (− 1.18; 0.84)	− 1.13 (− 1.63; 0.76)	1.25 (− 0.6; 5.31)	1.49 (− 0.88; 5.79)
	M	7.25 (1.58; 15.72)	5.52 (3.33; 8.19)	− 0.19 (− 1.13; 1.67)	− 0.9 (− 1.6; − 0.32)	1.49 (0.56; 2.61)	1.69 (− 0.36; 5.81)
Colon, Rectum & Anus	F	5.13 (2.74; 11.09)	5.05 (3.28; 8.76)	0.67 (0.01; 2.11)	0.46 (0.25; 1.04)	1.8 (0.24; 2.93)	2.79 (0.49; 5.07)
	M	5.89 (3.62; 9.46)	5.78 (3.87; 8.56)	2.08 (1.24; 2.71)	1.34 (0.93; 2.14)	2.5 (1.47; 3.33)	3.11 (1.64; 5.8)
Stomach	F	2.85 (− 1.07; 8.75)	1.75 (0.58; 5.77)	− 2.79 (− 3.59; − 2.13)	− 2.84 (− 3.29; − 1.88)	− 2.32 (− 2.69; − 0.19)	−1.07 (− 2.78; 1.76)
	M	1.66 (0.35; 7.35)	1.82 (0.41; 5.31)	− 3.12 (− 3.69; − 2.47)	−2.95 (− 3.37; − 2.44)	−2.67 (− 3.09; − 1.98)	−1.27 (− 2.94; 1.49)
Pancreas	F	8.79 (3.81; 14.82)	5.33 (2.36; 11.24)	0.85 (0.32; 2.44)	0.9 (0.44; 1.23)	2.23 (0.79; 3.95)	2.5 (0.89; 6.11)
	M	7.94 (1.67; 17.86)	5.64 (3.61; 9.54)	1.02 (0.28; 1.8)	1.03 (0.33; 1.17)	1.67 (0.13; 4.83)	2.38 (0.86; 6.67)
Lung	F	3.22 (1.75; 5.82)	5.18 (3.67; 7.23)	1.18 (0.59; 1.66)	1.39 (0.76; 2.02)	1.1 (0.05; 1.89)	2.02 (0.94; 4.62)
	M	2.6 (1.09; 6.45)	4.15 (1.9; 5.45)	− 0.9 (−1.47; 0.16)	− 0.91 (− 1.42; − 0.47)	0.3 (− 0.19; 0.61)	0.5 (− 0.91; 3.77)
Breast	F	6.89 (2.91; 17.76)	4.62 (2.91; 7.83)	0.29 (− 0.87; 1.32)	0.58 (− 0.17; 0.96)	2.08 (0.68; 3.82)	2.32 (0.38; 5.52)
Prostate	M	5.42 (3.39; 13.19)	5.11 (3.16; 7.04)	− 0.23 (− 1.28; 1.08)	− 0.26 (− 0.86; 0.25)	0.95 (0.44; 1.63)	1.52 (− 0.15; 5.1)
Cervical	F	3.1 (− 0.12; 6.43)	1.93 (0.02; 5.53)	− 3.01 (− 3.83; − 1.59)	−2.44 (− 2.75; − 1.54)	−2.37 (− 2.8; − 0.96)	− 0.97 (− 2.71; 2.09)

In the *Southeast region*, trends behaved differently. APCs were mostly stationary in the overall assessment of cancer mortality, and in the assessment of some specific types, as head and neck cancer (both sexes), lung and prostate cancer in men. Median APC values ranged from − 3.12% for stomach cancer to 2.08% for colorectal cancer in males (Table 1). The variation of APCs across intermediate regions was less pronounced than in the North and Northeast regions (Fig. 1). In the *South region*, most of the trends were decreasing, with APC median values ranging from − 2.95% (stomach cancer in males) to 1.39% (lung cancer in females) (Table 1). As in the Southeast, the variation of trends of cancer mortality across intermediate regions was reduced compared to the North and the Northeast. The *Center-West region* had predominantly increasing trends of mortality, except for stomach and cervical cancer, which were mostly decreasing in the intermediate regions. As in the South and Southeast, the variety of trends was less pronounced than in the North and Northeast macro-regions. Median APCs in the region ranged from − 2.67% for stomach cancer in males to 2.50% (the yearly increase of deaths) for colorectal cancer in males (Table 1). The Federal District was the intermediate region with the steepest decreasing trend for all cancer mortality in both sexes (− 1.46% [− 1.73%;-1.18%]) (Additional file 1: Table S5).

**Fig. 2:**
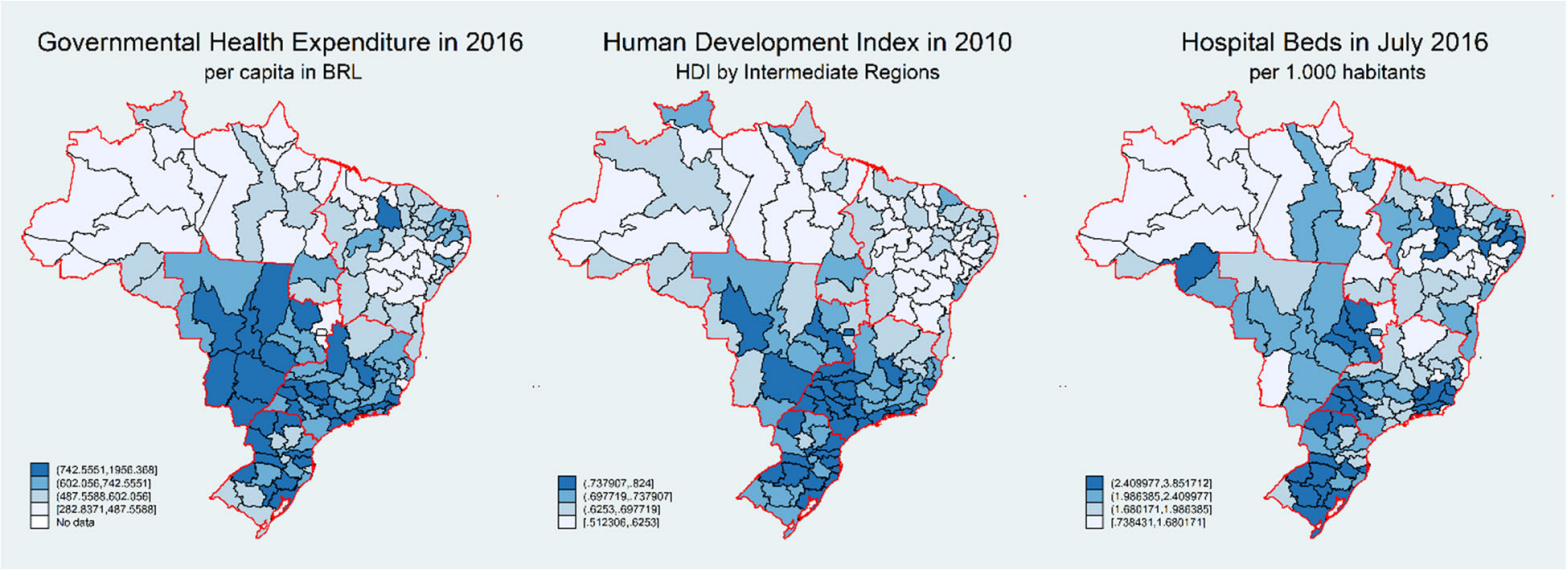
Health expenditure (per capita), human development, and hospital beds (per 1000 inhabitants) by intermediate regions

Cancer mortality trends in intermediate regions are associated with human development index and the provision of health resources. In general, the North and Northeast macro-regions mostly encompass impoverished intermediate regions; these regions also have a lower per capita government spending in health, and a reduced provision of hospital beds (Fig. 2). Overall and type-specific rates were mainly on the increase in the North and Northeast, in contrast to the remaining regions, which had a more similar profile of stationary and decreasing trends for many cancer types.

Median APCs for all cancers and some specific types correlated negatively with human development and health resources (Table 2). Regions with higher human development had decreasing trends of mortality, and progressively higher increase in trends occurred in areas with gradually lower human development index. Gradients were also evident in the assessment of health spending and hospital beds. Regions with a lower provision of health resources had a higher median APC. The assessment of p for trend corroborated that all associations were significant. The Human Development Index – HDI was negatively correlated with APC for all cancers and many specific types when stratified by macro-region; however, p for trends were most significant in the Northeast and the Southeast, which are the most populated regions in the country. This result is likely due to the lower number of intermediate areas and a more similar HDI profile in the remaining regions (Additional file 1: Table S6).

**Table 2:** Trends (annual percent change) of cancer mortality. Mean APC by type of cancer, sex, and quartiles of government health expenditure, hospital beds, and human development index. Brazil, 1996–2016

		Per Capita Gov Health Expenditure					Hospital beds, per 1000					Human Development Index				
	Sex	1st qtl	2nd qtl	3rd qtl	4th qtl	R(1)	1st qtl	2nd qtl	3rd qtl	4th qtl	R(1)	1st qtl	2nd qtl	3rd qtl	4th qtl	R(1)
All cancers	F	4.47	1.75	0.09	−0.50	− 0.49(2)	2.93	1.24	1.00	0.69	−0.37(2)	3.45	1.83	0.93	−0.27	−0.84(2)
	M	4.88	2.63	0.22	−0.62	−0.38(2)	3.60	1.51	1.25	0.80	−0.42(2)	3.99	2.31	1.22	−0.25	−0.87(2)
Head & Neck	F	12.25	3.43	0.51	−0.58	−0.38(2)	8.21	2.54	3.23	1.75	−0.37(3)	8.97	4.21	2.67	0.13	−0.66(2)
	M	11.11	4.73	1.14	−0.88	− 0.39(2)	8.49	2.61	2.52	2.56	−0.32(4)	9.92	3.42	3.29	−0.19	−0.63(2)
Colon, Rectum & Anus	F	9.19	3.41	1.85	0.46	−0.42(2)	6.52	3.35	2.92	2.19	−0.38(2)	6.97	4.14	3.08	0.96	−0.74(2)
	M	7.88	4.69	2.83	1.61	−0.47(2)	6.35	3.92	3.55	3.22	−0.33(2)	6.71	4.43	3.99	2.04	−0.68(2)
Stomach	F	7.73	0.64	−1.99	−2.90	−0.43(2)	4.44	0.27	−0.32	−1.11	−0.37(2)	5.57	1.54	−1.12	−2.55	−0.71(2)
	M	5.62	1.54	−2.24	−3.17	−0.47(2)	4.30	−0.53	−0.96	−1.13	− 0.36(2)	4.81	0.99	−1.23	−2.83	−0.71(2)
Pancreas	F	11.95	3.87	2.69	0.79	−0.42(2)	9.08	3.77	4.42	2.11	−0.41(2)	9.25	5.38	3.82	1.16	−0.65(2)
	M	10.89	5.34	1.57	0.95	−0.47(2)	8.82	3.98	3.25	2.76	−0.40(2)	9.61	4.98	3.26	1.20	−0.72(2)
Lung	F	7.67	3.14	1.09	1.22	−0.43(2)	5.28	3.24	2.41	2.25	−0.32(3)	6.28	3.63	2.31	1.09	−0.63(2)
	M	5.93	2.44	−0.21	−1.11	−0.43(2)	4.42	1.18	0.94	0.56	−0.38(2)	4.65	2.31	1.01	−0.71	−0.75(2)
Breast	F	11.82	3.19	1.99	−0.06	−0.39(2)	8.75	2.40	3.47	2.42	−0.31(3)	10.21	4.13	2.23	0.72	−0.56(2)
Prostate	M	8.89	4.55	0.79	−0.75	− 0.48(2)	7.29	2.05	2.67	1.51	−0.38(2)	8.52	3.55	1.85	−0.16	−0.71(2)
Cervical	F	5.98	1.96	−1.71	−3.01	−0.41(2)	4.96	−0.41	−0.67	− 0.62	−0.30(3)	6.13	0.75	−0.87	−2.53	−0.58(2)

(1) R = Pearson correlation.

(2) *P* for trend < 0.001

(3) *P* for trend = 0.001

(4) *P* for trend = 0.002

## Discussion

This study described cancer trends for all cancers combined and for major cancer groups in all intermediate regions of Brazil. Cancer mortality trends were increasing in the Northeast and North, whereas they were predominantly decreasing or stationary in the remaining macro-regions. This pattern reflects differences in human development and the provision of health care resources across the regions. Additionally, the variation of mortality trends was more pronounced in the North and Northeast than in the remaining regions, which showed a more similar epidemiologic profile. The results also pointed out to a steeper decrease in cancer mortality in areas with higher HDI even within specific macro-regions.

Barbosa et al. [21] reported comparable results and predicted that the decrease of all cancer mortality in the Southeast and South would result in an overall decreasing trend for the country by the year 2030. A literature review on cancer care in Kenya, Brazil and the US discussed disparities in outcomes and concluded that, despite having a well-implemented universal healthcare system, Brazil lacks advanced technologies and fails in providing equal access to the population, especially in inland areas [22]. The findings are consistent with the “Fundamental Causes Theory,” which states that there is an association between socioeconomic conditions and health status. Individuals with higher financial resources, education, favorable social connections, social status, and power would have better conditions to care for their health, and a lower risk for any disease. Conversely, individuals subjected to material deprivation would be more susceptible to the conditions and decisions that lead to an early decline in health, as well as the lower access to adequate care when afflicted by any disease [23, 24].

*Lung cancer* mortality started to decline in some countries around 1980, but the reduction among Brazilian men only began in the 2000s, after the adoption of anti-tobacco policies [25]. Silva et al. [26] reported differences of cancer mortality trends in state capitals and smaller municipalities, underscoring that trends were on the increase or leveled off among women in all regions. However, this previous study was not comprehensive of all Brazilian regions and missed critical differences in inland areas. Pelotas (state of Rio Grande do Sul), for instance, is an inland municipality with a high provision of health resources and human development. Its intermediate region had the sixth-highest decrease in lung cancer mortality in the country.

*Head and neck* cancer mortality trends ranked slightly higher for males than females. However, women living in the North region had the highest median APC in the country, concurrently with the highest variance across intermediate regions. Other authors have previously reported the poorer profile of trends for head and neck cancer mortality in the North and Northeast regions [7]. Some studies suggested that deaths by head and neck are preventable by early diagnosis and effective treatment; this subject is still a matter of controversies in the literature [27, 28]. The reduction of head and neck cancer in the more affluent regions may reflect, in part, the reduction of incidence that followed the reduction of the tobacco epidemics. The expansion of public dental services in Brazil, which occurred in the last decades, may have also contributed. In line with these hypotheses, Rocha [29] reported the association of lower mortality rates for oral cancer with public health funding and healthcare coverage.

The increasing trend of *colorectal cancer* mortality in all regions for both sexes is consistent with previous reports [28, 30]. This rise is likely mainly attributable to dietary patterns, especially meat consumption and lack of physical activity [31, 32]. However, these factors may not explain differences across the regions. Chow et al. [33] observed that, in the US, rural patients with colon cancer were more likely to have a late diagnosis and lower access to proper treatment. Furthermore, Rollet et al. [34] assessed if social deprivation and geographical access were mediating the influence of comorbidities and treatment on the rise of colon cancer mortality. They discarded the influence of comorbidities and confirmed geographical disparities in each step of the treatment. Therefore, we believe that higher increasing trends may reflect the lack of health infrastructure in poorer intermediate regions.

*Breast cancer* is the most common type of cancer in women in Brazil. Carioli et al. [35] assessed data provided by the Pan-American Health Organization to predict breast cancer mortality in the Americas and concluded that the trend was stationary in Brazil. This result eludes essential differences across the regions and is not supported by results reported here. Furthermore, the absent correction for underreporting and misclassification may have influenced their findings. Other studies, however, have agreed that breast cancer mortality is on the increase in the country [3, 26]. Breast cancer mortality is amenable to reduction by early diagnosis [36]. National screening programs in Brazil rely heavily on the infrastructure of the health system, and availability of services varies across regions and municipalities, long-waiting queues and delay in diagnosis may occur [37]. Patients that depend solely on the public health system are twice as likely to receive a stage III breast cancer diagnosis compared to those covered by private health insurance in Brazil [38]. Although the WHO recommends mammography screenings in upper-middle-income countries [39]; the inadequate health infrastructure has been consistently reported as an obstacle to providing screenings for the general population, and appropriate assistance for breast cancer patients in Brazil [37, 40, 41]. We noticed that intermediate regions with decreasing trends in breast cancer also had a decrease for other cancer types, which suggests that the availability of centers specialized in cancer treatment may contribute to the control of breast cancer.

*Prostate cancer* is the second most common cause of cancer deaths in men in Brazil. Previous studies already reported the poorer epidemiologic profile of prostate cancer mortality in the North and Northeast macro-regions [6, 42], consistent with our findings. Silva et al. [43] reported an inverse correlation between prostate cancer mortality and deaths by ill-defined causes, thus concluding that the recent improvement of mortality information in poorer regions may have influenced the assessment of trends. The contribution of screening in reducing prostate cancer mortality is uncertain; however, some studies suggested that the screening has no tangible impact at the population level [44, 45]. Braga et al. [42] attributed the rise in prostate cancer mortality to the process of population aging and regional disparities in access to healthcare. Other studies reported that having a regular physician and private health insurance was associated with a lower probability of being diagnosed in a metastatic stage [46, 47]. This finding is consistent with our results of a poorer evolution in prostate cancer deaths in intermediate regions with the reduced provision of health resources and low human development index.

*Cervical cancer* mortality differs across the country’s intermediate regions. In the North and Northeast, only some intermediate regions containing state capitals, and the regions of Gurupi in the North, and Iguatu on the Northeast had decreasing trends. However, in the South, Southeast, and Center-West regions, trends were decrescent or stationary. Barbosa et al. [48] has already reported regional disparities in cervical cancer mortality in Brazil. The overall reduction of cervical cancer mortality in Brazil and Latin America has been associated with the improvement of socioeconomic conditions [49]. Expanded coverage of public services of healthcare may play a role in reducing cervical cancer mortality. Still, women covered by the private health care system have higher chances of undergoing cervical cancer screenings [46, 50]. Lourenço et al. [51] stated that the varying availability of screening programs and healthcare infrastructure cannot explain disparities in late diagnosis of cervical cancer and that misconceptions about the Papanicolau test are a significant barrier against screening in low-income populations. Additionally, the quality of cytological tests appears to vary across the country. Discacciati et al. [52] observed that Maceió, a city in the Northeast region, had proportionally twice as many samples rejected than the city of Rio de Janeiro, in the Southeast. The authors argue that the lower quality of cytopathological exams in Maceió may have increased the number of false-negative results. Such factors can prevent early diagnosis and delay the delivery of care, giving rise to disparities in cervical cancer mortality across regions.

The overall decline of *stomach cancer* mortality in Brazil contrasts with those in the North and Northeast macro-regions, which were predominantly increasing. Consistent with our findings, Giusti et al. [53] reported higher APCs for males than for females in the whole country. Stomach cancer has a low survival rate; its reduction in Brazil and Latin-American countries is attributable to improvements in sanitation and food safety, both factors that reduce the risk of *H. pylori* infection [27]. Impoverished areas in the country, especially in rural zones, lack the necessary infrastructure to prevent this type of infection [54]. Practical nutritional advice is one of the objectives of the Family Health Program [55], a program whose coverage has increased continually since its creation in 1990.

*Pancreatic cancer* is increasing in the whole country, except for Uberlândia (Minas Gerais), in the Southeast region, which had a significantly decreasing trend for women. No previous study assessed trends of pancreatic cancer mortality across the Brazilian regions. Souza et al. [5] described patterns of incidence and lethality in the country and reported increasing trends for all age groups and a poorer profile in deprived areas. Pancreatic cancer is relatively infrequent; we cannot rule out that our analysis may not have been sensitive enough to detect trends in some intermediate regions, thus classifying them as stationary due to the lack of statistical power of the assessment. Like lung cancer, pancreatic cancer is considered one of the most lethal types of cancer, with less than 5% of individuals surviving more than 10 years after diagnosis [56]. Therefore, regional disparities of trends in both lung and pancreatic cancer are likely to be due to improvements in diagnosis and quality of the information provided by death certificates, with a lower contribution from the provision of healthcare.

Increasing trends of cancer mortality in less developed areas may have been influenced by an increase in the quality of the health information system over the years, mainly for the older individuals, whose cause of death is less extensively reported. This is the main study limitation, which we tried to attenuate by redistributing deaths by ill-defined causes and garbage codes based on methods built on literature reviews and extensive field investigation by the Global Burden of Disease Study [15]. Although the overall quality of mortality information improved since 1996 [57], death by ill-defined causes reaches up to ranked 13.7% of all deaths in the state of Bahia, and up to 20.0% at the intermediate region of Paulo Afonso, both in the Northeast region. Another limitation of the study is the use of a single APC to characterize the trend. Trends that are stationary in our results may have started decreasing only recently after years of steady increases. We choose to not focus of those shifts and calculate a single APC for the trend due to the large number of trends analyzed, however, we acknowledge that this would add important information about the historical pattern of cancer mortality in the country. The creation of new intermediate regions in 2017 did not represent a study limitation, because we could aggregate the data redistributing information related to each municipality to the correspondent intermediate region.

## Conclusion

Intermediate regions at the North and Northeast had more and higher increasing trends of overall and type-specific cancer mortality. These increasing trends can overburden their already fragile health infrastructure, with fewer resources than the remaining regions of the country. In addition to a lower provision of healthcare, these regions also suffer reduced human development. This study depicted the geographic association between trends of cancer mortality and government health expenditure, per-capita hospital beds and the human development index graphically; however, a more detailed analysis is necessary to explain how health services and programs interact with cancer mortality. Also, regional differences in access to private healthcare contribute to cancer mortality must be explored further. Regulatory authorities should implement health surveillance to identify areas with increasing trends of cancer mortality. They should also consider that mortality trends may be driven by the lack of access to healthcare not only in each municipality but also in its surrounding municipalities. Appropriate planning of healthcare provision can revert the ongoing increasing trends of mortality by major cancer groups in the poorer regions of Brazil.

## Supplementary information

**Additional file 1: Table S1.** APC by intermediate region and cancer group, NORTH region 1996-2016. **Table S2.** APC by intermediate region and cancer group, NORTHEAST region 1996-2016. **Table S3.** APC by intermediate region and cancer group, SOUTHEAST region 1996-2016. **Table S4.**. APC by intermediate region and cancer group, SOUTH region 1996-2016. **Table S5.** APC by intermediate region and cancer group, CENTER-WEST region 1996-2016. **Table S6.** Pearson correlation of Human Development Index and APC by cancer type and macro-region.

## Data Availability

The datasets analyzed during the current study are available in the Brazilian Ministry of Health repository, http://www2.datasus.gov.br/ and in the Atlas of Human Development in Brazil repository, http://atlasbrasil.org.br/2013/en/
