# Supplementary material for: Describing mortality trends for major cancer sites in 133 intermediate regions of Brazil and an ecological study of its causes
Source: BMC Cancer. 2019 Oct 11;19:940. doi: 10.1186/s12885-019-6184-1 (PMC6788078; doi:10.1186/s12885-019-6184-1)
Supplement: Supplementary file 1 — Additional file 1: Table S1. APC by intermediate region and cancer group, NORTH region 1996-2016. Table S2. APC by intermediate region and cancer group, NORTHEAST region 1996-2016. Table S3. APC by intermediate region and cancer group, SOUTHEAST region 1996-2016. Table S4.. APC by intermediate region and cancer group, SOUTH region 1996-2016. Table S5. APC by intermediate region and cancer group, CENTER-WEST region 1996-2016. Table S6. Pearson correlation of Human Development Index and APC by cancer type and macro-region. [file 12885_2019_6184_MOESM1_ESM.docx]

**Article Title:** Describing mortality trends for major cancer sites in 133 intermediate regions of Brazil and an ecological study of its causes

**Journal:** BMC Cancer

**Authors:**

Alessandro Bigoni^1^ ( [alebigoni@usp.br](mailto:alebigoni@usp.br) ) ( [ORCID: 0000-0002-2483-3299](https://orcid.org/0000-0002-2483-3299) )

José Leopoldo Ferreira Antunes^1^ ( [leopoldo@usp.br](mailto:leopoldo@usp.br) ) ( [ORCID: 0000-0003-3972-9723](https://orcid.org/0000-0003-3972-9723) )

Elisabete Weiderpass^2,3^ ( [weiderpasse@iarc.fr](mailto:weiderpasse@iarc.fr) ) ( [ORCID: 0000-0003-2237-0128](https://orcid.org/0000-0003-2237-0128) )

Kristina Kjærheim^3^ ( [Kristina.Kjaerheim@kreftregisteret.no](mailto:Kristina.Kjaerheim@kreftregisteret.no) ) ([ORCID: 0000-0003-0691-3735](https://orcid.org/0000-0003-0691-3735))

**Corresponding author:**

**Name:** Alessandro Bigoni

**Mailing Address:** Universidade de São Paulo, Faculdade de Saúde Pública, Departamento de Epidemiologia. Av. Dr. Arnaldo 715, Pacaembu, Sao Paulo, SP – Brasil CEP: 01246-904

**E-mail Address:** [alebigoni@usp.br](mailto:alebigoni@usp.br)

**Table S1: APC by intermediate region and cancer group, NORTH region 1996-2016**

| **N** | **All Cancers** | | | **Head & Neck** | | | **Colon & Rectum** | | | **Stomach** | | | **Pancreas** | | | **Lung** | | | **Breast** | **Prostate** | **Cervix** |
| --- | --- | --- | --- | --- | --- | --- | --- | --- | --- | --- | --- | --- | --- | --- | --- | --- | --- | --- | --- | --- | --- |
|  | F | M | FM | F | M | FM | F | M | FM | F | M | FM | F | M | FM | F | M | FM | F | M | F |
| Porto Velho* | -0.74 [-1.66; 0.18] | 0.48 [-0.65; 1.62] | 0.07 [-1.02; 1.17] | -1.78 [-4.76; 1.3] | 1.68 [-1.09; 4.53] | 1.06 [-1.29; 3.47] | 0.49 [-1.78; 2.81] | 1.5 [-0.22; 3.24] | 0.74 [-0.58; 2.08] | -3.64[-6.46; -0.74] | -1.75[-2.79; -0.69] | -2.3[-3.13; -1.47] | -0.68 [-3.26; 1.97] | 5.56 [1.04; 10.29] | 2.23 [0.51; 3.98] | -2.14 [-4.2; -0.03] | -1.78 [-2.74; -0.8] | -2.02 [-3.11; -0.92] | 6.65 [0.98; 12.64] | 0.06 [-1.08; 1.21] | -0.12 [-2.39; 2.21] |
| Ji-Paraná | 0.94 [-0.11; 1.99] | 1.68 [1.3; 2.05] | 1.36 [0.8; 1.92] | -3.92 [-11.41; 4.21] | 0.61 [-0.98; 2.23] | -0.58 [-2.49; 1.37] | 3.87 [1.56; 6.23] | 3.13 [0.88; 5.43] | 3.41 [1.57; 5.29] | -0.77[-3.1; 1.62] | -1.26[-2.65; 0.14] | -1.2[-2.11; -0.27] | 3.04 [-1.92; 8.25] | 0.1 [-4.77; 5.22] | 0.17 [-3.13; 3.58] | 2.68 [0.3; 5.12] | 2.03 [0.41; 3.67] | 2.23 [0.8; 3.69] | 3.64 [1.46; 5.87] | 3.39 [2.01; 4.78] | 0.55 [-1.35; 2.49] |
| Rio Branco* | 1.06 [0.17; 1.96] | 1.47 [0.69; 2.25] | 1.28 [0.7; 1.85] | 3.35 [-3.98; 11.25] | 1.58 [-2.67; 6.02] | 1.19 [-1.74; 4.2] | 5.07 [1.05; 9.25] | 5.24 [1.25; 9.39] | 3.97 [1.23; 6.79] | -2.2[-5.72; 1.45] | 0.55[-4.01; 5.32] | -0.34[-4.47; 3.96] | 2.17 [-4.93; 9.81] | 10.32 [2.14; 19.17] | 4.28 [-2.4; 11.43] | 2.69 [-1.18; 6.72] | 1.8 [-2.52; 6.32] | 2.25 [-1.74; 6.39] | 2.61 [-3.65; 9.26] | 0.75 [-2.12; 3.71] | -0.63 [-5.43; 4.42] |
| Cruzeiro do Sul | 4.32 [2.54; 6.14] | 5.75 [3.34; 8.22] | 4.89 [3.45; 6.35] | 22.82 [8.72; 38.75] | 23.47 [9.45; 39.28] | 17.91 [6.57; 30.46] | 20.08 [14.27; 26.18] | 9.9 [3.09; 17.16] | 13.66 [8.04; 19.57] | 12.85[-4.4; 33.21] | 17.83[5.22; 31.95] | 11.97[3.75; 20.83] | 6.15 [-8.61; 23.3] | 24.59 [13.01; 37.37] | 12.14 [-0.85; 26.82] | 7.53 [-1.69; 17.61] | 6.45 [0.38; 12.88] | 3.86 [1.4; 6.38] | 20.31 [9.74; 31.91] | 13.55 [3.28; 24.84] | 5.58 [-5.56; 18.03] |
| Manaus* | 0.46 [-0.16; 1.09] | 0.56 [-0.03; 1.15] | 0.54 [0.01; 1.07] | 0.33 [-1.07; 1.74] | 1.26 [0.22; 2.3] | 1.05 [0.11; 2] | 0.81 [-0.5; 2.14] | 3.62 [2.31; 4.95] | 1.95 [1.14; 2.77] | -0.88[-1.79; 0.04] | 0.35[-0.75; 1.46] | -0.07[-1.06; 0.92] | 4.55 [2.57; 6.58] | 1.67 [0.68; 2.68] | 2.75 [1.94; 3.57] | 0.72 [-0.4; 1.85] | -2.05 [-2.66; -1.43] | -1.17 [-1.74; -0.59] | 1.53 [-0.44; 3.53] | 1.95 [0.81; 3.1] | 1.03 [-0.51; 2.58] |
| Tefé | 6.23 [2.64; 9.93] | 4.91 [1.63; 8.31] | 5.33 [2.07; 8.69] | 14.06 [-1.8; 32.5] | 8.1 [-0.1; 16.99] | 5.63 [0.38; 11.17] | 12.97 [-3.5; 32.24] | 8.7 [1.58; 16.33] | 8.26 [-1.67; 19.2] | 15.96[1.23; 32.83] | 6.12[1.34; 11.13] | 6.54[2.04; 11.23] | 19.29 [5.02; 35.5] | 25.17 [19.07; 31.57] | 24.09 [14.47; 34.53] | 4.63 [-0.24; 9.74] | 5.22 [2.57; 7.94] | 4.08 [1.69; 6.52] | 26.38 [16.5; 37.1] | 13.64 [3.28; 25.05] | 20.11 [5.48; 36.78] |
| Lábrea | 4.45 [2.23; 6.72] | 3.17 [1.52; 4.85] | 3.56 [2.13; 5.01] | 17.34 [3.49; 33.04] | 15.87 [4.37; 28.63] | 13.61 [3.45; 24.77] | 11.09 [2.89; 19.93] | -1.66 [-7.81; 4.9] | 2.74 [-2.44; 8.21] | 8.67[-4.25; 23.34] | 6.46[-9.4; 25.1] | 2.5[-2.83; 8.13] | 18.96 [9.22; 29.57] | 21.44 [7.59; 37.09] | 21.18 [10.11; 33.37] | 5.06 [-2.81; 13.56] | 2.61 [-0.34; 5.66] | 2.44 [-0.98; 5.97] | 17.82 [-2.13; 41.83] | 5.35 [-1.8; 13.01] | 3.51 [-0.82; 8.04] |
| Parintins | 4.32 [2.17; 6.51] | 5.21 [3.72; 6.72] | 4.58 [2.74; 6.45] | 3.25 [-6.77; 14.34] | 15.72 [4.05; 28.71] | 9.14 [3.17; 15.45] | 8.44 [-1.21; 19.02] | 5.87 [-1.15; 13.38] | 4.73 [1.4; 8.17] | 8.75[0.51; 17.65] | 7.35[4.65; 10.12] | 6.1[2.58; 9.74] | 22.05 [8.99; 36.67] | 17.04 [0.3; 36.59] | 20.11 [6.24; 35.78] | 14.27 [2.99; 26.8] | 14.9 [2.72; 28.53] | 11.8 [2.95; 21.4] | 29.31 [7.52; 55.52] | 13.19 [3.54; 23.74] | 6.56 [4.16; 9.01] |
| Boa Vista* | -0.21 [-1.32; 0.92] | -0.55 [-1.84; 0.76] | -0.42 [-1.46; 0.63] | -0.27 [-4.09; 3.7] | -1.94 [-4.64; 0.83] | -1.72 [-3.72; 0.31] | 3.58 [-3.02; 10.63] | 1.64 [-0.64; 3.97] | 0.6 [-1.86; 3.12] | -1.52[-8.53; 6.03] | 1.2[-6.37; 9.38] | 0.13[-6.66; 7.41] | 7.02 [-3.21; 18.32] | -2.08 [-4.94; 0.87] | 0.61 [-2.4; 3.7] | 1.75 [-1.86; 5.49] | -0.94 [-5; 3.3] | -0.44 [-2.44; 1.6] | 0.78 [-3.56; 5.31] | 0.63 [-3.14; 4.55] | -0.29 [-3.79; 3.33] |
| Rorainópolis - Caracaraí | 0.69 [-2.49; 3.97] | 2.83 [0.89; 4.81] | 1.2 [-0.98; 3.44] | 37.83 [-0.89; 91.68] | 22.12 [9.8; 35.82] | 19.41 [6.38; 34.02] | 16.1 [-0.58; 35.58] | 5.91 [-3.2; 15.88] | 8.19 [0.79; 16.13] | 13.41[-6.41; 37.43] | 7.45[-2.13; 17.96] | 5.37[-3.27; 14.78] | 9.67 [-12.36; 37.25] | 1.46 [-16.66; 23.51] | -0.33 [-18.26; 21.53] | 27.08 [12.68; 43.32] | 7.93 [-1.55; 18.33] | 2.48 [-4.25; 9.69] | 63.93 [-2.36; 175.22] | 27.77 [6.29; 53.58] | 23.78 [-4.47; 60.38] |
| Belém* | -0.09 [-0.41; 0.23] | -0.25 [-0.61; 0.11] | -0.18 [-0.51; 0.14] | 1.29 [-0.07; 2.67] | -0.68 [-1.48; 0.13] | -0.25 [-1; 0.51] | 0.8 [-0.51; 2.12] | 1.73 [0.85; 2.62] | 1.19 [0.34; 2.06] | -1.07[-2.34; 0.23] | -1.12[-1.65; -0.59] | -1.12[-1.8; -0.43] | 0.89 [-0.71; 2.52] | 1.34 [0.1; 2.6] | 1.11 [-0.02; 2.24] | -0.17 [-1.05; 0.71] | -2.09 [-2.57; -1.6] | -1.51 [-1.92; -1.11] | 1.23 [0.61; 1.85] | 0.56 [-0.44; 1.57] | -1.66 [-2.69; -0.61] |
| Castanhal | 5.76 [4.01; 7.53] | 6.74 [4.44; 9.09] | 5.87 [3.74; 8.05] | 19.19 [5.69; 34.43] | 8.97 [6.36; 11.64] | 9.93 [6.53; 13.44] | 9.22 [7.02; 11.46] | 10.35 [5.65; 15.26] | 9.56 [6.51; 12.69] | 3.14[-1.35; 7.84] | 7.54[3.46; 11.78] | 5.56[1.98; 9.27] | 22.4 [12.11; 33.63] | 14.93 [8.04; 22.25] | 14.69 [8.59; 21.14] | 6.72 [3.74; 9.79] | 4.57 [2.69; 6.48] | 5 [3.07; 6.96] | 8.58 [6.04; 11.17] | 9.82 [6.95; 12.76] | 6.43 [3.96; 8.96] |
| Marabá | 3.64 [1.67; 5.65] | 3.75 [2.61; 4.9] | 3.71 [2.12; 5.33] | 9.43 [4.94; 14.12] | 5.12 [1.71; 8.63] | 6.1 [3.15; 9.13] | 4.09 [2.44; 5.76] | 4.45 [1.88; 7.09] | 3.7 [2.24; 5.18] | 2.2[-1.53; 6.08] | 2.13[-1.06; 5.43] | 1.89[-0.88; 4.75] | 14.82 [0.94; 30.6] | 2.99 [-4.7; 11.29] | 3.88 [-0.57; 8.52] | 5.82 [3.56; 8.13] | 4.59 [1.61; 7.66] | 4.92 [3.07; 6.8] | 7.12 [3.44; 10.93] | 5.1 [3.42; 6.81] | 2.68 [0.94; 4.45] |
| Redenção | 2.88 [0.69; 5.12] | 3.24 [2.19; 4.31] | 3 [1.74; 4.29] | 9.32 [4.27; 14.61] | 10.93 [2.41; 20.15] | 2.99 [-0.88; 7.01] | 6.34 [0.63; 12.39] | 7.91 [2.78; 13.29] | 6.94 [1.85; 12.29] | 5.12[-1.93; 12.68] | -1.87[-6.74; 3.26] | -1.07[-4.15; 2.1] | 28.63 [16.66; 41.82] | 11.16 [0.82; 22.55] | 17.37 [9.61; 25.68] | 4.73 [1.19; 8.39] | 2.6 [-0.64; 5.94] | 3.21 [0.69; 5.79] | 8.13 [-6.39; 24.9] | 12.25 [2.53; 22.88] | 3.53 [-1.3; 8.59] |
| Santarém | 3.59 [2.68; 4.5] | 3.46 [2.75; 4.18] | 3.53 [2.8; 4.25] | 7.87 [1.28; 14.89] | 1.5 [-2.14; 5.27] | 1.97 [-1.94; 6.04] | 2.74 [-0.55; 6.14] | 5.95 [2.58; 9.42] | 4.12 [1.97; 6.31] | 0.16[-4.62; 5.18] | 1.1[-3.54; 5.96] | 0.98[-3.72; 5.91] | 4.76 [-8.84; 20.39] | 5.34 [-0.8; 11.86] | 4.52 [-3.13; 12.78] | 2.69 [-3.69; 9.5] | 1.83 [-1.85; 5.66] | 2.5 [-1.63; 6.81] | 6.28 [2.86; 9.8] | 4.67 [2.15; 7.25] | 2.29 [0.68; 3.93] |
| Altamira | 2.96 [1.45; 4.49] | 4.3 [2.27; 6.38] | 3.62 [2.25; 5.01] | 19.8 [3.46; 38.71] | 6.4 [-2.91; 16.61] | 8.68 [-1.07; 19.4] | 10.57 [6.08; 15.26] | 8.3 [-0.71; 18.13] | 7.4 [2.17; 12.89] | 10.04[1.49; 19.3] | 4.3[-0.29; 9.1] | 3.84[-0.25; 8.1] | 9.15 [-5.68; 26.3] | 17.97 [5.87; 31.44] | 14.49 [1.67; 28.94] | 0.1 [-4.18; 4.58] | 6.59 [-0.2; 13.84] | 2.8 [-1.32; 7.1] | 17.76 [2.74; 34.98] | 9.3 [-0.05; 19.53] | 5.91 [0.14; 12.01] |
| Breves | 5.18 [3.49; 6.88] | 4.7 [2.92; 6.52] | 4.79 [3.31; 6.3] | 18.99 [13.63; 24.6] | 26.81 [10.7; 45.25] | 18.51 [9.43; 28.35] | 11.14 [3.18; 19.72] | 11.47 [2.71; 20.98] | 10.73 [4.29; 17.57] | 14.33[0.1; 30.57] | 7.89[1.06; 15.17] | 4.03[0.2; 8.02] | 10.6 [-6.63; 31.01] | 21.83 [11.86; 32.68] | 12.84 [2.36; 24.39] | 12.06 [4.08; 20.65] | 8.11 [3.7; 12.71] | 8.62 [4.46; 12.95] | 11.7 [2.17; 22.11] | 18.16 [8.57; 28.6] | 7.84 [5.59; 10.15] |
| Macapá* | -0.6 [-1.93; 0.74] | -0.17 [-1.81; 1.49] | -0.33 [-1.74; 1.1] | -1.3 [-6.37; 4.04] | 2.12 [-2.59; 7.07] | 1.28 [-2.72; 5.45] | 1.08 [-3.37; 5.74] | 4.36 [-1.52; 10.58] | 2.22 [-0.27; 4.77] | -2.65[-6.05; 0.87] | -1.19[-2.89; 0.54] | -1.61[-3.45; 0.26] | 8.44 [1.33; 16.04] | -1.16 [-5.68; 3.59] | 0.28 [-2.26; 2.89] | -1.61 [-4.88; 1.78] | -2.01 [-3.4; -0.6] | -2.08 [-3.39; -0.75] | 9.14 [2.46; 16.25] | 3.81 [2.22; 5.42] | -2.34 [-4.96; 0.37] |
| Oiapoque - Porto Grande | 2.21 [-2.3; 6.93] | 8.16 [0.17; 16.78] | 3.89 [-0.02; 7.96] | -18.12 [-42.75; 17.11] | 25.35 [3.76; 51.44] | 13.92 [-5.43; 37.22] | -7.64 [-21.33; 8.43] | 15.08 [1.39; 30.6] | 11.61 [-0.66; 25.41] | -9.51[-24.31; 8.18] | 27.07[2.58; 57.42] | 19.19[2.28; 38.9] | -1.04 [-16.45; 17.21] | 17.86 [2.85; 35.07] | 24.47 [12.47; 37.74] | 2.84 [-11.8; 19.92] | 18.87 [2.39; 38.01] | 9.33 [-0.66; 20.34] | -8.29 [-36.56; 32.58] | 34.31 [9.95; 64.07] | 49.67 [14.73; 95.25] |
| Palmas* | 3.16 [1.29; 5.07] | 3.1 [1.36; 4.86] | 3.06 [1.23; 4.93] | 10.15 [-4.34; 26.83] | 10.36 [0.78; 20.85] | 9.7 [-0.29; 20.69] | 11.17 [4.85; 17.88] | 12.24 [5.29; 19.65] | 11.01 [5.01; 17.35] | 2.55[-1.99; 7.3] | 0.44[-1.96; 2.89] | 0.97[-1.31; 3.31] | 9.55 [1.76; 17.94] | 11.24 [1.7; 21.66] | 8.27 [2.77; 14.07] | 3.09 [0.05; 6.21] | 1.53 [-1.93; 5.12] | 1.9 [-0.41; 4.26] | 5.58 [3.19; 8.01] | 4.8 [3.3; 6.33] | 4.51 [1.41; 7.7] |
| Araguaína | 2.73 [1.49; 3.99] | 4.63 [2.75; 6.56] | 3.69 [2.2; 5.2] | 11.03 [1.31; 21.69] | 6.09 [3.28; 8.98] | 6.39 [3.22; 9.65] | 5.18 [1.92; 8.55] | 4.1 [1.47; 6.79] | 4.3 [2.36; 6.27] | 4.99[-0.48; 10.75] | 1.87[0.1; 3.66] | 2.09[0.34; 3.86] | 3.81 [1.49; 6.18] | 2.43 [-1.47; 6.48] | 3.02 [1.46; 4.61] | 3.34 [0.44; 6.34] | 5.5 [2.47; 8.62] | 4.49 [1.72; 7.34] | 5.64 [3.7; 7.63] | 5.74 [3.08; 8.47] | 2.09 [-0.22; 4.46] |
| Gurupi | 1.57 [0.37; 2.78] | 3.05 [2.2; 3.92] | 2.25 [1.5; 3] | 1.72 [-4.85; 8.74] | 13.85 [3.61; 25.1] | 6.86 [2.18; 11.76] | 3.61 [-1.26; 8.73] | 9.46 [3.89; 15.33] | 5.26 [1.58; 9.07] | 3.96[-5.93; 14.9] | 1.46[-1.05; 4.04] | 1.01[-1.83; 3.92] | 9.25 [-0.59; 20.06] | 4.29 [-7.24; 17.25] | 2.43 [-2.37; 7.46] | 4.25 [0.02; 8.67] | 1.09 [-0.93; 3.16] | 1.99 [-0.04; 4.05] | 2.91 [-0.09; 6.01] | 5.49 [2.98; 8.07] | -2.06 [-3.01; -1.1] |

* Intermediate regions containing state capitals ; Increasing trend, Decreasing trend, Stationary trend.

**Table S2: APC by intermediate region and cancer group, NORTHEAST region 1996-2016**

| **NE** | **All Cancers** | | | **Head & Neck** | | | **Colon & Rectum** | | | **Stomach** | | | **Pancreas** | | | **Lung** | | | **Breast** | **Prostate** | **Cervix** |
| --- | --- | --- | --- | --- | --- | --- | --- | --- | --- | --- | --- | --- | --- | --- | --- | --- | --- | --- | --- | --- | --- |
|  | F | M | FM | F | M | FM | F | M | FM | F | M | FM | F | M | FM | F | M | FM | F | M | F |
| São Luís* | 1.66 [0.88; 2.44] | 2.36 [1.82; 2.9] | 1.98 [1.29; 2.67] | 4.39 [2.12; 6.7] | 3.79 [2.13; 5.47] | 3.79 [2.36; 5.24] | 3.08 [1.97; 4.2] | 4.96 [3.4; 6.53] | 3.78 [3.24; 4.33] | 1.76[0.06; 3.48] | 1.98[1.02; 2.95] | 1.93[0.96; 2.9] | 4.43 [2.55; 6.34] | 3.61 [1.81; 5.43] | 3.85 [2.63; 5.09] | 2.86 [1.97; 3.77] | 0.55 [-0.56; 1.67] | 1.33 [0.32; 2.35] | 3.29 [2.71; 3.86] | 3.16 [1.44; 4.91] | 0.06 [-1.34; 1.47] |
| Santa Inês - Bacabal | 8.95 [6; 11.98] | 8.77 [5.76; 11.87] | 8.85 [5.51; 12.29] | 17.95 [11.09; 25.23] | 9.96 [4.82; 15.34] | 10.92 [6.03; 16.03] | 10.78 [4.74; 17.16] | 9.11 [5.63; 12.69] | 9.01 [5.89; 12.22] | 22.45[13.33; 32.3] | 6.87[3.2; 10.68] | 8.36[4.88; 11.96] | 20.19 [8.72; 32.86] | 24.3 [14.79; 34.59] | 20.08 [10.09; 30.98] | 19.75 [10; 30.36] | 10.99 [7.48; 14.61] | 14.1 [8.29; 20.24] | 9.42 [5.43; 13.56] | 11.08 [8.99; 13.22] | 7.4 [5.01; 9.86] |
| Caxias | 5.04 [3.52; 6.58] | 4.89 [3.82; 5.96] | 4.95 [3.75; 6.16] | 21.99 [14.51; 29.97] | 9.14 [6.3; 12.05] | 10.05 [7.36; 12.81] | 13.07 [4.49; 22.35] | 3.76 [1.89; 5.66] | 6.6 [5.01; 8.22] | 13.24[3.71; 23.65] | 4.4[1.6; 7.27] | 4.32[2.8; 5.86] | 20.49 [12.47; 29.09] | 7.76 [1.32; 14.61] | 7.64 [4.78; 10.57] | 6.26 [4.18; 8.38] | 5.93 [3.66; 8.25] | 5.67 [4.18; 7.18] | 9.41 [7.14; 11.72] | 7.04 [4.46; 9.68] | 4.55 [1.61; 7.57] |
| Presidente Dutra | 8.06 [4.57; 11.66] | 7.79 [3.99; 11.73] | 7.57 [3.84; 11.44] | 17.91 [13.33; 22.67] | 22.46 [7.15; 39.96] | 14.42 [8.76; 20.37] | 11.9 [5.6; 18.58] | 13.62 [3.56; 24.67] | 9.53 [4.87; 14.4] | 15.93[9.36; 22.89] | 12.7[3.63; 22.56] | 7.14[3.52; 10.89] | 27.19 [17.89; 37.23] | 11.82 [1.6; 23.07] | 15.48 [6; 25.8] | 8.67 [3.68; 13.9] | 10.44 [6.02; 15.04] | 8.66 [5.82; 11.59] | 24.33 [13.18; 36.59] | 17.3 [10.42; 24.61] | 10.03 [3.62; 16.84] |
| Imperatriz | 8.23 [5.16; 11.4] | 7.37 [5.26; 9.53] | 7.76 [5.24; 10.33] | 17.04 [8.85; 25.86] | 9.9 [6.72; 13.18] | 9.8 [8.38; 11.23] | 13.49 [7.82; 19.45] | 9.19 [6.39; 12.05] | 10.34 [7.29; 13.46] | 15.71[6.93; 25.22] | 8.93[5.76; 12.18] | 8.53[6.37; 10.72] | 12.74 [7.67; 18.04] | 9.54 [4.94; 14.34] | 7.49 [4.55; 10.5] | 10.83 [6.71; 15.1] | 7.33 [4.63; 10.09] | 8.25 [5.75; 10.82] | 12.29 [6.55; 18.35] | 10.07 [5.03; 15.35] | 8.56 [4.68; 12.58] |
| Teresina* | 2.17 [1.82; 2.52] | 3.33 [2.17; 4.5] | 2.77 [2.02; 3.52] | 5.93 [3.16; 8.77] | 4.31 [3.65; 4.98] | 4.46 [3.57; 5.36] | 4.38 [3.25; 5.52] | 5.77 [4.36; 7.19] | 5.07 [4.37; 5.77] | 3.2[1.42; 5] | 3.19[1.11; 5.31] | 3.18[1.31; 5.08] | 2.1 [0.42; 3.8] | 3.16 [1.8; 4.54] | 2.81 [1.88; 3.75] | 3.22 [1.68; 4.79] | 1.93 [0.55; 3.34] | 2.36 [1.22; 3.52] | 4.56 [3.08; 6.07] | 2.1 [0.94; 3.28] | -0.25 [-1.48; 0.99] |
| Parnaíba | 5.82 [3.38; 8.32] | 5.92 [3.86; 8.03] | 5.92 [3.69; 8.21] | 5.96 [0.99; 11.18] | 9.51 [4.15; 15.13] | 8.03 [3.6; 12.66] | 13.95 [6.56; 21.86] | 9.16 [5.33; 13.13] | 10.5 [5.47; 15.77] | 9.75[-2.8; 23.92] | 4.87[1.35; 8.52] | 4.4[1.47; 7.4] | 9.03 [2.05; 16.48] | 5.2 [0.09; 10.56] | 4.54 [1.98; 7.17] | 5.35 [3.46; 7.27] | 3.04 [0.99; 5.13] | 3.87 [2.02; 5.76] | 11.16 [2.56; 20.48] | 5.1 [2.27; 8] | 4.35 [-0.42; 9.34] |
| Picos | 7.8 [4.13; 11.6] | 7.28 [4.04; 10.62] | 7.53 [4.07; 11.1] | 20.18 [7.41; 34.47] | 12.01 [5.26; 19.19] | 12 [5.8; 18.58] | 14.25 [9.14; 19.6] | 7.87 [5.9; 9.88] | 9.84 [7.57; 12.15] | 4.99[0.88; 9.26] | 5.31[1.75; 8.99] | 5.04[1.6; 8.6] | 18.57 [2.34; 37.37] | 13.12 [1.12; 26.55] | 14.57 [2.06; 28.61] | 5.55 [2.57; 8.63] | 6.16 [1.78; 10.73] | 5.96 [2.49; 9.54] | 14.93 [5.87; 24.77] | 16.4 [4.59; 29.53] | 6.58 [2.21; 11.14] |
| São Raimundo Nonato | 9.46 [3.5; 15.76] | 9.68 [2.39; 17.48] | 9.45 [2.78; 16.55] | 21.36 [11.26; 32.38] | 55.41 [20; 101.28] | 23.93 [11.8; 37.38] | 15.75 [3.02; 30.06] | 20.22 [3.53; 39.61] | 14.68 [4.19; 26.22] | 21.58[10.08; 34.27] | 20.61[1.36; 43.5] | 19.02[2.67; 37.97] | 2.82 [-11.54; 19.5] | 19.04 [-3.98; 47.57] | 10.42 [-5.77; 29.39] | 15.28 [6.55; 24.73] | 16.91 [-1.69; 39.04] | 14.86 [1.31; 30.23] | 24.57 [8.3; 43.27] | 20.36 [7.12; 35.24] | 30.46 [9.17; 55.9] |
| Corrente - Bom Jesus | 5.89 [2.04; 9.88] | 5.88 [3.01; 8.83] | 5.36 [2.22; 8.6] | 19.46 [3.12; 38.4] | 3.53 [-10.63; 19.93] | 8.2 [-2.56; 20.15] | 13.62 [1.17; 27.6] | 12.98 [8.08; 18.1] | 9.1 [4.15; 14.28] | 24.87[-5.32; 64.69] | 21.93[11.75; 33.05] | 17.59[5.48; 31.09] | 13.17 [-16.07; 52.59] | 3.35 [-16.09; 27.29] | 4.75 [-9.54; 21.3] | 4.39 [-2.64; 11.94] | 10.5 [7.11; 13.99] | 8.26 [4.56; 12.09] | 13.68 [-1.94; 31.79] | 6.93 [1.27; 12.9] | 11.86 [3.49; 20.91] |
| Floriano | 5.64 [2.92; 8.44] | 6.12 [3.41; 8.91] | 5.75 [2.79; 8.78] | 22.8 [1.48; 48.6] | 35.21 [13.5; 61.06] | 18.23 [8.09; 29.33] | 14.37 [7.45; 21.73] | 18.32 [11.04; 26.08] | 15.42 [9.35; 21.84] | 10.81[4.4; 17.61] | 7.35[-3.97; 20] | 5.96[-1.92; 14.46] | 27.19 [13.92; 42.01] | 17.09 [4.2; 31.57] | 18.35 [7.77; 29.97] | 7.23 [2.68; 11.99] | 11.36 [5.01; 18.1] | 7.48 [4.32; 10.74] | 5.22 [0.13; 10.57] | 5.57 [2.07; 9.18] | 6.69 [1.31; 12.35] |
| Fortaleza* | 0.21 [-0.31; 0.74] | 0.45 [-0.08; 0.99] | 0.35 [-0.16; 0.86] | -0.63 [-1.55; 0.3] | 1.17 [0.12; 2.22] | 0.76 [-0.12; 1.64] | 2.12 [1.11; 3.14] | 2.95 [2.27; 3.65] | 2.55 [1.76; 3.34] | -1.29[-1.81; -0.77] | -1.42[-2.16; -0.68] | -1.39[-1.9; -0.87] | 2.36 [1.33; 3.4] | 2.38 [1.18; 3.6] | 2.29 [1.41; 3.17] | 2.19 [1.76; 2.63] | 0.5 [-0.21; 1.22] | 1.15 [0.62; 1.68] | 0.88 [0.53; 1.22] | -0.22 [-0.74; 0.3] | -0.99 [-1.57; -0.4] |
| Quixadá | 2.66 [0.83; 4.52] | 3.1 [1.53; 4.7] | 3 [1.23; 4.81] | -1.29 [-4.05; 1.54] | 1.67 [0.17; 3.19] | 0.57 [-0.85; 2] | 4.35 [1.32; 7.46] | 6.06 [3.47; 8.72] | 5.32 [2.66; 8.05] | 1.44[-0.98; 3.92] | 0.93[-0.98; 2.87] | 1.24[-0.85; 3.38] | 3.96 [1.34; 6.63] | 5.1 [0.86; 9.52] | 3.97 [1.72; 6.26] | 5.6 [3.89; 7.34] | 6.01 [3.9; 8.16] | 5.78 [4.18; 7.4] | 3.31 [2.05; 4.57] | 1.56 [-0.15; 3.3] | 2.01 [-2.19; 6.38] |
| Iguatu | 0.75 [-0.5; 2.03] | 2.2 [0.91; 3.49] | 1.52 [0.3; 2.75] | -2.8 [-6.7; 1.25] | 0.3 [-1.92; 2.57] | -0.6 [-3.15; 2.02] | 2.77 [0.07; 5.53] | 2.41 [0.56; 4.29] | 2.39 [0.64; 4.17] | -2.21[-3.9; -0.5] | -0.49[-2.74; 1.81] | -1.17[-2.64; 0.32] | 12.77 [2.35; 24.25] | 12.22 [7.12; 17.57] | 10.94 [5.92; 16.2] | 7.59 [4.25; 11.04] | 5.11 [3.03; 7.23] | 5.74 [3.9; 7.6] | 1.01 [-1.55; 3.63] | 1.42 [-0.64; 3.53] | -3.09 [-5.52; -0.59] |
| Juazeiro do Norte | 3.71 [2.72; 4.71] | 3.57 [2.32; 4.83] | 3.65 [2.51; 4.8] | 5.31 [2.13; 8.59] | 4.46 [2.19; 6.79] | 4.67 [2.32; 7.07] | 5.06 [3.3; 6.85] | 5.28 [3.86; 6.73] | 5.08 [4.15; 6.02] | 0.76[-0.97; 2.51] | 1.67[-0.11; 3.48] | 1.41[-0.16; 3] | 4.74 [2.92; 6.59] | 8.68 [2.6; 15.12] | 5.34 [3.09; 7.65] | 5.13 [3.5; 6.79] | 3.77 [2.08; 5.48] | 4.35 [3.01; 5.71] | 4.54 [3.26; 5.84] | 3.92 [1.9; 5.98] | 3.51 [0.12; 7.02] |
| Crateús | 3.53 [1.86; 5.23] | 4.04 [2.1; 6.01] | 3.83 [2.06; 5.62] | 3.26 [-3.97; 11.03] | 6.53 [2.41; 10.81] | 5.23 [1.97; 8.6] | 7.8 [5.51; 10.15] | 9.33 [6.95; 11.77] | 8.29 [6.82; 9.77] | 1.25[-1.52; 4.1] | 2.27[0.12; 4.47] | 1.86[0.08; 3.67] | 12.33 [3.64; 21.74] | 4.21 [-4.75; 14.01] | 4.77 [2.75; 6.82] | 9.13 [7.23; 11.05] | 7.02 [3.49; 10.67] | 7.72 [5.09; 10.42] | 2.7 [1.18; 4.24] | 1.91 [-0.17; 4.03] | 1.57 [-0.46; 3.63] |
| Sobral | 2.39 [0.92; 3.88] | 3.28 [1.3; 5.31] | 2.87 [1.09; 4.68] | 3.04 [-0.93; 7.18] | 3.14 [1.27; 5.04] | 2.96 [0.81; 5.15] | 5.23 [1.72; 8.87] | 4.95 [3.07; 6.87] | 5 [2.45; 7.62] | 1.48[-0.45; 3.44] | 1.04[-0.31; 2.4] | 1.16[-0.16; 2.5] | 4.21 [0.59; 7.96] | 3.71 [2.39; 5.05] | 3.87 [1.53; 6.27] | 6.22 [4.14; 8.34] | 4.25 [0.96; 7.64] | 5.51 [2.56; 8.55] | 2.91 [1.66; 4.17] | 4.29 [2.57; 6.04] | 0.02 [-1.83; 1.91] |
| Natal* | 0.83 [0.44; 1.23] | 1.98 [1.47; 2.5] | 1.42 [0.97; 1.86] | 1.61 [0.48; 2.75] | 2.9 [1.91; 3.9] | 2.57 [1.77; 3.37] | 3.03 [2.44; 3.62] | 2.29 [1.01; 3.59] | 2.67 [2.1; 3.25] | 0.13[-0.86; 1.13] | 0.21[-0.3; 0.71] | 0.14[-0.31; 0.6] | 1.38 [0.33; 2.43] | 2.69 [1.03; 4.38] | 2.04 [1.51; 2.58] | 3.38 [1.77; 5.02] | 1.09 [-0.53; 2.74] | 1.74 [0.39; 3.12] | 0.97 [-0.41; 2.37] | 2.94 [1.65; 4.23] | -1.66 [-2.69; -0.61] |
| Caicó | 2.87 [1.91; 3.83] | 4.73 [3.33; 6.15] | 3.74 [2.64; 4.84] | 11.63 [-0.14; 24.79] | 6.37 [3.93; 8.86] | 5.58 [3.12; 8.09] | 3.49 [-0.91; 8.08] | 5.56 [2.09; 9.14] | 4.31 [0.93; 7.79] | -0.12[-3.42; 3.29] | 1.19[-1.72; 4.2] | 0.54[-1.51; 2.63] | 8.72 [2.66; 15.12] | 14.69 [7.84; 21.97] | 8.72 [3.03; 14.72] | 4.5 [2.08; 6.97] | 5.45 [2.42; 8.57] | 4.85 [3.38; 6.33] | 5.7 [2.95; 8.52] | 6.44 [2.81; 10.21] | -0.64 [-4.59; 3.48] |
| Mossoró | 3.04 [2.16; 3.93] | 3.45 [2.6; 4.3] | 3.25 [2.48; 4.02] | 5.79 [2.23; 9.48] | 7.09 [4.88; 9.34] | 6.42 [4.27; 8.6] | 5.05 [2.4; 7.77] | 4.9 [3.01; 6.83] | 4.89 [3.06; 6.76] | 0.58[-0.5; 1.67] | 1.44[0.01; 2.89] | 1.11[-0.02; 2.26] | 5.71 [1.49; 10.1] | 3.36 [0.57; 6.23] | 4.51 [2.92; 6.13] | 6.51 [5.47; 7.56] | 4.18 [2.13; 6.28] | 5.06 [3.74; 6.41] | 5.77 [3.64; 7.96] | 4.05 [2.82; 5.31] | -0.45 [-2.19; 1.33] |
| João Pessoa* | 2.34 [1.36; 3.34] | 3.52 [2.18; 4.87] | 2.94 [1.73; 4.15] | 4.79 [1.69; 7.98] | 4.32 [1.52; 7.2] | 4.24 [1.84; 6.7] | 4.79 [3.27; 6.33] | 5.8 [3.3; 8.35] | 5.19 [3.41; 7] | 1.41[-0.32; 3.18] | 2.74[0.37; 5.17] | 2.07[-0.09; 4.27] | 6.11 [1.31; 11.13] | 4.66 [2.88; 6.47] | 4.97 [2.61; 7.38] | 3.86 [3.12; 4.61] | 1.89 [0.65; 3.15] | 2.55 [1.59; 3.51] | 2.52 [0.66; 4.42] | 6.06 [3.59; 8.58] | 2.56 [0.58; 4.58] |
| Campina Grande | 4.8 [2.16; 7.52] | 6.23 [3.57; 8.96] | 5.49 [2.87; 8.18] | 9.22 [4.49; 14.16] | 11.32 [8.64; 14.06] | 10.66 [7.76; 13.64] | 5.85 [0.51; 11.47] | 8.56 [2.85; 14.59] | 6.39 [1.34; 11.7] | 4.77[1.22; 8.45] | 6.95[4.93; 9] | 6.09[3.56; 8.68] | 10.27 [5.01; 15.79] | 9.35 [5.69; 13.14] | 8.7 [6.37; 11.09] | 5.33 [2.13; 8.63] | 4.92 [2.3; 7.61] | 5.08 [2.31; 7.92] | 5.36 [3.16; 7.61] | 9.14 [5.45; 12.95] | 6.39 [4.07; 8.76] |
| Patos | 4.78 [2.13; 7.5] | 5.53 [3.83; 7.26] | 5.25 [3.13; 7.41] | 6.7 [3.58; 9.92] | 6.21 [4.2; 8.25] | 6.28 [4.52; 8.07] | 13.83 [6.53; 21.63] | 10.18 [7.69; 12.73] | 10.54 [7.26; 13.92] | 1.39[-0.58; 3.4] | 4.33[2.32; 6.39] | 2.93[1.43; 4.44] | 11.1 [5.18; 17.36] | 9.67 [2.95; 16.84] | 7.49 [2.78; 12.42] | 8.38 [4.71; 12.17] | 5.44 [2.83; 8.13] | 6.55 [4.28; 8.87] | 4.67 [1.88; 7.53] | 8.79 [5.21; 12.49] | 4.63 [0; 9.47] |
| Sousa - Cajazeiras | 4.32 [2.69; 5.98] | 4.34 [2.56; 6.15] | 4.33 [2.63; 6.05] | 6.85 [0.22; 13.92] | 8.19 [3.73; 12.86] | 6.81 [4.38; 9.3] | 5.71 [1.86; 9.71] | 2.86 [-0.21; 6.03] | 3.85 [1.35; 6.42] | 3.98[1.95; 6.05] | 5.34[-0.83; 11.9] | 3.89[0.44; 7.46] | 11.24 [3.14; 19.97] | 20.96 [11.73; 30.97] | 13.81 [5.91; 22.3] | 6.99 [2.17; 12.05] | 4.41 [-0.2; 9.23] | 5.37 [1.35; 9.55] | 8.01 [3.65; 12.56] | 9.71 [3.28; 16.55] | 5.53 [2.81; 8.32] |
| Recife* | -0.35 [-0.5; -0.19] | 0.34 [0.08; 0.61] | 0.04 [-0.14; 0.22] | 0.08 [-0.8; 0.98] | 0.73 [0.12; 1.35] | 0.61 [0.06; 1.16] | 0.69 [-0.02; 1.41] | 3.02 [2.42; 3.61] | 1.78 [1.19; 2.36] | -0.36[-0.74; 0.01] | -0.04[-0.61; 0.54] | -0.17[-0.54; 0.21] | 1.13 [0.61; 1.64] | 0.88 [0.24; 1.52] | 1 [0.61; 1.39] | 1.94 [1.38; 2.5] | -0.37 [-0.86; 0.13] | 0.32 [0; 0.65] | 0.54 [0.22; 0.87] | 0.18 [-0.32; 0.67] | -3.14 [-3.45; -2.83] |
| Caruaru | 1.91 [0.76; 3.08] | 3.36 [2.49; 4.25] | 2.76 [1.73; 3.8] | 4.35 [1.56; 7.23] | 3.33 [2.44; 4.22] | 3.39 [2.21; 4.58] | 3.34 [2.27; 4.43] | 5.49 [4.34; 6.66] | 4.31 [3.35; 5.27] | 1.85[0.42; 3.3] | 2.05[0.78; 3.33] | 1.99[0.86; 3.13] | 5.82 [0.18; 11.79] | 3.67 [2.28; 5.09] | 3.88 [1.73; 6.08] | 4.3 [3.14; 5.48] | 2.84 [1.87; 3.81] | 3.34 [2.87; 3.82] | 2.29 [0.73; 3.88] | 3.9 [2.27; 5.56] | 0.37 [-0.46; 1.22] |
| Serra Talhada | 2.05 [1.44; 2.67] | 2.23 [1.02; 3.46] | 2.08 [1.18; 2.98] | 4.39 [0.07; 8.9] | 2.45 [-0.48; 5.47] | 2.57 [0.43; 4.76] | 4.93 [2.46; 7.46] | 7.57 [2.91; 12.44] | 5.36 [3.26; 7.51] | 1.91[0.43; 3.42] | 1.49[-1.45; 4.52] | 1.54[-0.52; 3.65] | 1.72 [-1.65; 5.2] | 0.93 [-1.11; 3.02] | 1.35 [-0.78; 3.53] | 4.48 [2.33; 6.67] | 4.12 [1.27; 7.05] | 3.74 [1.17; 6.38] | 3.77 [1.6; 5.99] | 3.21 [0.39; 6.1] | 0.24 [-1.86; 2.39] |
| Petrolina | 3.18 [2.18; 4.19] | 3.89 [2.29; 5.53] | 3.5 [2.07; 4.94] | 0.05 [-2.38; 2.54] | 4.98 [1.84; 8.21] | 3.36 [1.41; 5.34] | 5.53 [3.51; 7.6] | 6.16 [4.47; 7.87] | 5.72 [4.55; 6.9] | -0.04[-2.08; 2.04] | 0.41[-1.22; 2.06] | 0.22[-0.87; 1.31] | 1.3 [-1.5; 4.17] | 7.29 [5.5; 9.12] | 4.3 [2.61; 6.01] | 4.62 [3.22; 6.05] | 4.11 [2.45; 5.8] | 4.33 [3.06; 5.61] | 3.56 [2.19; 4.94] | 3.57 [1.38; 5.82] | 2.63 [0.4; 4.92] |
| Maceió* | 0.46 [-0.17; 1.09] | 1.13 [0.65; 1.61] | 0.81 [0.42; 1.21] | 2.92 [0.42; 5.49] | 3.1 [2.14; 4.06] | 2.83 [2.01; 3.65] | 2.9 [0.65; 5.21] | 2.91 [1.71; 4.13] | 2.87 [1.67; 4.1] | -1.39[-3.31; 0.57] | 0.37[-0.94; 1.69] | -0.35[-1.56; 0.88] | 1.65 [-0.19; 3.52] | 3.2 [-0.01; 6.52] | 2.38 [0.41; 4.39] | 2.16 [-1.43; 5.89] | 0.88 [-2.09; 3.94] | 1.31 [-1.79; 4.51] | 2.8 [1.85; 3.77] | 2.7 [0.87; 4.57] | 0.09 [-1.1; 1.29] |
| Arapiraca | 4.48 [3.2; 5.77] | 5.33 [4.29; 6.38] | 4.96 [3.84; 6.08] | 9.03 [4.67; 13.56] | 7.24 [5.06; 9.47] | 7.46 [5.27; 9.7] | 7.11 [3.54; 10.81] | 7.66 [4.57; 10.83] | 7.32 [4.48; 10.23] | 3.09[-0.85; 7.19] | 4.18[1.7; 6.72] | 3.63[1.83; 5.47] | 4.59 [2.02; 7.23] | 7.73 [1.7; 14.11] | 5.81 [2.57; 9.14] | 4.86 [3.58; 6.15] | 3.77 [2.81; 4.73] | 4.18 [3.47; 4.89] | 6.09 [4.24; 7.97] | 6.79 [4.65; 8.97] | 1.85 [-0.6; 4.36] |
| Aracaju* | 0.31 [-0.1; 0.73] | 1.39 [0.55; 2.23] | 0.9 [0.3; 1.49] | 1.08 [-0.85; 3.05] | 3.65 [1.35; 6.01] | 3.04 [0.96; 5.17] | 3.19 [1.54; 4.87] | 3.81 [1.83; 5.83] | 3.35 [2.03; 4.68] | 0.92[-1.49; 3.39] | 0.32[-1; 1.64] | 0.45[-0.96; 1.88] | 1.78 [-0.23; 3.82] | 3.9 [1.79; 6.07] | 2.62 [1.4; 3.86] | -0.45 [-1.53; 0.64] | 0.1 [-1.21; 1.43] | -0.22 [-0.95; 0.52] | 3.08 [2.3; 3.87] | 2.41 [0.83; 4.01] | -1.33 [-2.64; -0.01] |
| Itabaiana | 5.24 [2.45; 8.1] | 5.88 [3.19; 8.64] | 5.5 [2.81; 8.27] | 18.36 [4.76; 33.72] | 10.03 [5.15; 15.14] | 10.65 [4.81; 16.81] | 8.76 [3.74; 14.01] | 8.49 [6.53; 10.48] | 8.25 [5.03; 11.58] | 8.51[2.46; 14.91] | 6.36[2.49; 10.37] | 5.68[2.25; 9.22] | 17.61 [7.35; 28.85] | 9.14 [2.25; 16.49] | 10.4 [4.28; 16.89] | 3.67 [1.21; 6.18] | 5.04 [2.08; 8.09] | 4.45 [2.43; 6.5] | 7.83 [4.67; 11.1] | 11.2 [4.84; 17.95] | 7.16 [2.37; 12.17] |
| Salvador* | -0.15 [-0.38; 0.09] | 0 [-0.26; 0.27] | -0.09 [-0.32; 0.14] | -1.68 [-2.51; -0.84] | -0.41 [-1; 0.18] | -0.65 [-1.13; -0.18] | 0.94 [0.25; 1.64] | 1.8 [1.15; 2.46] | 1.39 [0.83; 1.94] | -1.5[-2.61; -0.38] | -1.74[-2.89; -0.57] | -1.67[-2.66; -0.67] | 2.25 [1.09; 3.41] | 3.33 [2.09; 4.6] | 2.79 [1.84; 3.73] | 1.41 [0.66; 2.17] | -1.97 [-2.58; -1.35] | -1.02 [-1.39; -0.64] | 0.8 [-0.19; 1.8] | 0.02 [-0.31; 0.36] | -2.44 [-3.08; -1.78] |
| Santo Antônio de Jesus | 2.06 [1.46; 2.67] | 2.48 [2.13; 2.83] | 2.3 [1.97; 2.63] | 3.21 [1.56; 4.88] | 2.87 [1.84; 3.91] | 2.91 [1.96; 3.87] | 3.25 [1.9; 4.61] | 3.87 [2.51; 5.25] | 3.48 [2.54; 4.44] | 1.14[-2.26; 4.65] | 0.31[-0.85; 1.48] | 0.71[-0.34; 1.78] | 3.75 [0.94; 6.64] | 6.67 [4.4; 8.98] | 5.27 [3.17; 7.42] | 3.26 [0.74; 5.84] | 1.47 [-0.33; 3.29] | 2.04 [0.12; 4] | 3.54 [1.83; 5.28] | 3.54 [2.54; 4.55] | -0.47 [-1.66; 0.74] |
| Ilhéus – Itabuna | 2.07 [1.59; 2.54] | 3.22 [2.69; 3.75] | 2.71 [2.21; 3.21] | 2.04 [0.49; 3.6] | 3.88 [2.2; 5.58] | 3.2 [2.15; 4.26] | 3.28 [1.29; 5.32] | 3.68 [2.36; 5.01] | 3.25 [1.85; 4.68] | 0.37[-0.94; 1.7] | 0.22[-0.98; 1.45] | 0.33[-0.45; 1.12] | 4.43 [2.81; 6.08] | 4.41 [1.3; 7.61] | 4.28 [2.58; 6.01] | 3.94 [2.71; 5.19] | 1.61 [0.76; 2.46] | 2.38 [1.46; 3.31] | 3.49 [2.16; 4.83] | 5.12 [4.09; 6.16] | 1.04 [-0.55; 2.65] |
| Vitória da Conquista | 3.27 [2.63; 3.92] | 4.21 [2.98; 5.46] | 3.76 [2.84; 4.68] | 4.51 [1.75; 7.34] | 5.23 [3.53; 6.95] | 4.85 [2.98; 6.76] | 5.03 [3.65; 6.44] | 5.22 [3.28; 7.2] | 5.07 [3.59; 6.57] | 1.74[0.07; 3.44] | 1.24[-0.44; 2.96] | 1.21[-0.35; 2.79] | 4.13 [1.63; 6.7] | 3.65 [1.14; 6.22] | 3.52 [1.95; 5.11] | 5.07 [3.4; 6.76] | 3.28 [1.98; 4.6] | 3.83 [2.87; 4.79] | 6.09 [4.33; 7.89] | 5.61 [4.15; 7.1] | 1.59 [0.65; 2.54] |
| Guanambi | 3.87 [3.12; 4.62] | 3.49 [2.56; 4.44] | 3.61 [3.03; 4.2] | 5.73 [1.01; 10.67] | 6.22 [3.16; 9.37] | 5.68 [2.95; 8.48] | 3.14 [1.67; 4.64] | 5.67 [3.26; 8.13] | 4.36 [3.24; 5.48] | -0.55[-2.62; 1.58] | -1.16[-2.85; 0.57] | -0.9[-1.74; -0.05] | 1.75 [-1.19; 4.78] | 7.61 [0.43; 15.3] | 3.33 [-0.49; 7.3] | 10.39 [4.7; 16.39] | 3.65 [0.66; 6.73] | 4.79 [2.9; 6.71] | 5.04 [2.72; 7.42] | 3.6 [2.54; 4.68] | 4.49 [2.11; 6.94] |
| Barreiras | 1.18 [0.33; 2.04] | 3.16 [2.27; 4.06] | 2.24 [1.45; 3.03] | 11.72 [5.25; 18.59] | 6.45 [1.59; 11.54] | 7.14 [2.66; 11.81] | 7.39 [2.3; 12.72] | 8.96 [3.92; 14.24] | 6.59 [3.15; 10.13] | 5.77[-1.38; 13.44] | 0.56[-1.42; 2.58] | 1.21[-0.31; 2.74] | 5.68 [-1.77; 13.7] | 1.88 [-3.81; 7.91] | 1.04 [-1.38; 3.53] | 1.8 [-1.94; 5.69] | 2.67 [0.17; 5.23] | 1.82 [-0.18; 3.87] | 1.58 [-1.12; 4.36] | 6.71 [3.91; 9.57] | 0.23 [-2.88; 3.44] |
| Irecê | 4.17 [2.61; 5.75] | 4.68 [3.9; 5.47] | 4.46 [3.39; 5.54] | 4.88 [0.49; 9.46] | 6.42 [3.2; 9.74] | 5.59 [2.74; 8.52] | 8.4 [4.03; 12.97] | 7.85 [4.09; 11.74] | 7.41 [5.23; 9.64] | 3.91[0.13; 7.83] | 1.25[-1.17; 3.72] | 1.4[-1.26; 4.12] | 2.31 [-4.98; 10.16] | 7.57 [3.08; 12.25] | 3.41 [0.19; 6.73] | 7.97 [4.6; 11.45] | 4.29 [2.18; 6.44] | 5.22 [3.18; 7.3] | 8.05 [3.8; 12.48] | 5.47 [3.44; 7.53] | 6.07 [3.54; 8.66] |
| Juazeiro | 5.57 [4.54; 6.62] | 5.64 [4.76; 6.53] | 5.62 [4.9; 6.34] | 12.06 [5.54; 18.98] | 5.81 [3.87; 7.8] | 6.31 [4.46; 8.2] | 8.54 [5.21; 11.97] | 5.99 [2.75; 9.33] | 6.82 [5.26; 8.4] | 8.91[2.2; 16.05] | 3.36[1.08; 5.69] | 3.59[1.83; 5.39] | 7.73 [2.4; 13.34] | 6.08 [0.06; 12.46] | 5.42 [2.61; 8.31] | 11.49 [7.01; 16.15] | 4.36 [3.24; 5.49] | 6.05 [4.56; 7.57] | 7.08 [5.5; 8.68] | 8.33 [5.64; 11.1] | 2.94 [0.76; 5.16] |
| Paulo Afonso | 4.41 [2.79; 6.05] | 5.81 [4.84; 6.79] | 5.2 [4.24; 6.17] | 10.08 [3.39; 17.2] | 5.98 [3.99; 8] | 5.82 [3.89; 7.78] | 4.92 [1.78; 8.16] | 6.32 [4.23; 8.46] | 5.45 [3.18; 7.76] | 3.33[0.17; 6.59] | 8.84[2.48; 15.6] | 3.99[1.98; 6.05] | 6.35 [-1.54; 14.87] | 17.07 [3.76; 32.08] | 7.22 [3.19; 11.41] | 5.6 [2.99; 8.27] | 4.37 [2.25; 6.54] | 4.57 [3.59; 5.56] | 7.63 [5.26; 10.07] | 6.91 [2.6; 11.41] | 4.69 [2.17; 7.28] |
| Feira de Santana | 2.83 [2.37; 3.28] | 3.49 [2.83; 4.15] | 3.16 [2.6; 3.72] | 2.13 [-0.51; 4.83] | 3.75 [2.84; 4.68] | 3.25 [2.25; 4.27] | 4.83 [2.8; 6.9] | 4.56 [2.83; 6.32] | 4.76 [3.55; 5.98] | 1.33[-0.22; 2.91] | 0.58[-0.12; 1.28] | 0.8[0.14; 1.46] | 4.97 [2.3; 7.71] | 4.68 [2.82; 6.58] | 4.54 [3.34; 5.76] | 5.23 [3.72; 6.75] | 1.9 [0.74; 3.06] | 2.91 [2.26; 3.57] | 3.29 [2.35; 4.25] | 4.66 [2.94; 6.41] | 1.27 [0.11; 2.44] |

* Intermediate regions containing state capitals ; Increasing trend, Decreasing trend, Stationary trend.

**Table S3: APC by intermediate region and cancer group, SOUTHEAST region 1996-2016**

| **SE** | **All Cancers** | | | **Head & Neck** | | | **Colon & Rectum** | | | **Stomach** | | | **Pancreas** | | | **Lung** | | | **Breast** | **Prostate** | **Cervix** |
| --- | --- | --- | --- | --- | --- | --- | --- | --- | --- | --- | --- | --- | --- | --- | --- | --- | --- | --- | --- | --- | --- |
|  | F | M | FM | F | M | FM | F | M | FM | F | M | FM | F | M | FM | F | M | FM | F | M | F |
| Belo Horizonte* | -0.61 [-0.79; -0.43] | -0.62 [-0.84; -0.39] | -0.62 [-0.82; -0.43] | 0.65 [-0.25; 1.55] | 0.64 [0.16; 1.12] | 0.64 [0.21; 1.06] | 0.72 [0.29; 1.15] | 2.38 [1.84; 2.92] | 1.59 [1.2; 1.97] | -2.79[-3.51; -2.07] | -2.94[-3.34; -2.54] | -2.88[-3.18; -2.59] | 0.84 [-0.34; 2.03] | 0.86 [0.05; 1.68] | 0.84 [0.12; 1.57] | 0.38 [-0.14; 0.91] | -1.4 [-1.91; -0.88] | -0.9 [-1.24; -0.55] | 0.31 [-0.27; 0.9] | -1.33 [-2.4; -0.24] | -2.89 [-3.35; -2.42] |
| Montes Claros | 1.23 [0.9; 1.56] | 2.36 [1.89; 2.83] | 1.91 [1.57; 2.24] | 4.77 [1.49; 8.15] | 4.11 [2.24; 6.02] | 4.07 [2.14; 6.04] | 3.57 [1.19; 6] | 4.08 [2.68; 5.5] | 3.77 [2.33; 5.23] | 0.29[-2.09; 2.73] | -0.25[-1.4; 0.92] | -0.14[-1.14; 0.88] | 2.62 [-0.12; 5.42] | 1.31 [-1.95; 4.67] | 1.72 [-0.41; 3.9] | 2.3 [1.09; 3.52] | 1.18 [0.39; 1.97] | 1.47 [0.78; 2.17] | 2.75 [1.59; 3.92] | 4.29 [2.16; 6.46] | -2.95 [-4.12; -1.78] |
| Teófilo Otoni | 2.25 [1.44; 3.07] | 2.97 [1.63; 4.33] | 2.71 [1.65; 3.78] | 2.74 [-0.33; 5.91] | 3.84 [0.48; 7.3] | 3.61 [0.93; 6.35] | 2.95 [1.8; 4.11] | 2.55 [1.37; 3.73] | 2.75 [1.94; 3.56] | -0.65[-2.4; 1.13] | 0.1[-2.25; 2.51] | -0.1[-2.27; 2.11] | 0.4 [-2.93; 3.84] | 2.65 [0.37; 4.99] | 1.6 [-0.11; 3.34] | 3.46 [2.42; 4.5] | 1.94 [0.97; 2.93] | 2.44 [1.7; 3.19] | 4.29 [2.22; 6.4] | 4.58 [2.62; 6.57] | 1.76 [-0.42; 3.99] |
| Governador Valadares | 1.42 [0.52; 2.33] | 1.92 [0.83; 3.02] | 1.59 [0.64; 2.56] | 4.62 [-0.36; 9.84] | 4.99 [3.08; 6.94] | 4.86 [2.91; 6.86] | 4.26 [2.23; 6.32] | 4.21 [2.38; 6.07] | 3.99 [2.35; 5.65] | -0.13[-2.51; 2.3] | 0.23[-0.88; 1.35] | 0.08[-1.16; 1.34] | 2.44 [0.14; 4.8] | 3.22 [0.81; 5.69] | 2.53 [1.36; 3.73] | 1.18 [-0.82; 3.22] | 1.4 [-0.14; 2.97] | 1.31 [-0.27; 2.92] | 5.52 [3.68; 7.4] | 2.43 [0.66; 4.23] | 1.21 [-0.86; 3.32] |
| Ipatinga | 0.48 [0.07; 0.89] | 0.88 [0.43; 1.33] | 0.7 [0.3; 1.11] | -1.64 [-5.92; 2.83] | 2.71 [1.42; 4.01] | 1.76 [0.31; 3.24] | 3.04 [1.32; 4.78] | 3.88 [2.38; 5.4] | 3.3 [1.93; 4.69] | -2.13[-4.4; 0.2] | -2.47[-3.34; -1.59] | -2.39[-3.53; -1.24] | 3.72 [-1.49; 9.19] | 0.26 [-1.04; 1.58] | 0.88 [-1.08; 2.88] | 0.15 [-1.37; 1.69] | 0.44 [-0.75; 1.64] | 0.35 [-0.81; 1.52] | 4.23 [3.12; 5.35] | 1.48 [-0.11; 3.1] | 0.44 [-0.84; 1.74] |
| Juíz de Fora | 0.14 [-0.32; 0.61] | 0.21 [-0.58; 1.01] | 0.17 [-0.49; 0.84] | -0.6 [-1.87; 0.69] | -0.46 [-2.22; 1.34] | -0.58 [-2.21; 1.08] | 2.11 [1.04; 3.18] | 3.18 [2.37; 4] | 2.61 [1.89; 3.34] | -3.69[-4.99; -2.38] | -3.29[-4.59; -1.97] | -3.45[-4.7; -2.17] | 1.86 [0.57; 3.18] | 1.6 [0.43; 2.8] | 1.73 [0.59; 2.89] | 1.49 [1.04; 1.94] | 0.16 [-0.72; 1.05] | 0.6 [-0.05; 1.27] | 1.32 [0.46; 2.18] | 1.08 [-0.51; 2.68] | -0.86 [-1.73; 0.02] |
| Barbacena | 0.15 [-0.34; 0.63] | 0.28 [-0.5; 1.07] | 0.22 [-0.36; 0.81] | 0.13 [-2.81; 3.15] | 1.57 [-0.28; 3.45] | 1.17 [-0.49; 2.86] | 2.71 [1.21; 4.24] | 2.71 [1.58; 3.86] | 2.69 [1.65; 3.74] | -2.95[-4.39; -1.49] | -2.95[-4.36; -1.53] | -2.89[-4.34; -1.42] | 0.17 [-0.68; 1.02] | 0.08 [-2.17; 2.37] | 0.09 [-1.01; 1.21] | 2.34 [0.3; 4.42] | 0.84 [-0.8; 2.5] | 1.17 [-0.34; 2.71] | 0.8 [-0.41; 2.04] | 0.35 [-1.16; 1.87] | -0.44 [-1.35; 0.49] |
| Varginha | -0.75 [-1.07; -0.44] | -0.18 [-0.44; 0.08] | -0.42 [-0.65; -0.19] | 0.84 [-0.85; 2.56] | 1.67 [0.78; 2.56] | 1.55 [0.81; 2.29] | -0.73 [-1.76; 0.31] | 2.08 [1.3; 2.87] | 0.71 [0.18; 1.24] | -4.98[-5.66; -4.28] | -3.23[-4.44; -2] | -3.77[-4.71; -2.83] | 0.32 [-0.59; 1.24] | 1.8 [-0.03; 3.68] | 1 [0.11; 1.89] | 0.59 [-1.49; 2.72] | -0.2 [-1.17; 0.79] | -0.05 [-1.17; 1.09] | 0.32 [-0.53; 1.19] | -0.2 [-1.23; 0.83] | -3.65 [-5.11; -2.17] |
| Pouso Alegre | -0.64 [-1.14; -0.15] | -0.46 [-0.62; -0.29] | -0.53 [-0.79; -0.26] | -1.25 [-2.48; 0] | 0.15 [-1.36; 1.69] | -0.27 [-1.55; 1.02] | 0.08 [-1.04; 1.2] | 1.67 [0.79; 2.56] | 0.91 [0.18; 1.64] | -2.67[-3.59; -1.74] | -3.19[-4.78; -1.58] | -3.07[-4.28; -1.84] | 0.32 [-1.48; 2.15] | -0.67 [-2.72; 1.42] | -0.29 [-1.79; 1.24] | 0.24 [-1.43; 1.94] | -1.16 [-2.29; -0.01] | -0.66 [-1.85; 0.56] | -0.71 [-2.55; 1.16] | -0.23 [-0.92; 0.47] | -3.39 [-5.36; -1.38] |
| Uberaba | -0.2 [-0.79; 0.39] | 0.05 [-0.23; 0.33] | -0.07 [-0.43; 0.29] | -0.49 [-2.15; 1.2] | 0 [-1.71; 1.74] | -0.19 [-1.48; 1.12] | 0.17 [-1.04; 1.41] | 2.21 [0.86; 3.57] | 1.24 [0.16; 2.34] | -2.74[-5.69; 0.31] | -2.6[-4.17; -1.01] | -2.78[-4.27; -1.28] | 3.68 [-0.41; 7.93] | 1.1 [-0.45; 2.67] | 1.75 [-0.21; 3.75] | 1.66 [0.88; 2.46] | -0.26 [-1.08; 0.56] | 0.37 [-0.3; 1.05] | -0.87 [-2.37; 0.66] | 0.32 [-0.64; 1.3] | -4.29 [-5.77; -2.78] |
| Uberlândia | -1 [-1.5; -0.5] | -0.58 [-1.06; -0.1] | -0.76 [-1.16; -0.35] | -0.02 [-1.03; 1] | 0.1 [-1.48; 1.71] | 0.05 [-1.38; 1.5] | 0.01 [-0.88; 0.9] | 2.49 [1.54; 3.44] | 1.28 [0.48; 2.09] | -4.1[-5.78; -2.39] | -3.97[-5; -2.92] | -3.94[-5.05; -2.82] | -1.36 [-2.65; -0.05] | -0.28 [-1.62; 1.08] | -0.75 [-1.64; 0.14] | 0.09 [-1.23; 1.42] | -0.96 [-1.72; -0.19] | -0.59 [-1.3; 0.13] | 0.32 [-0.71; 1.36] | -1.43 [-2.38; -0.47] | -4.56 [-5.39; -3.72] |
| Patos de Minas | 0.29 [-0.16; 0.74] | 1.02 [0.43; 1.62] | 0.62 [0.21; 1.02] | 3.25 [-0.18; 6.79] | 2.38 [0.38; 4.43] | 2.36 [0.41; 4.35] | 2.81 [0.93; 4.72] | 2.62 [0.32; 4.97] | 2.59 [1.14; 4.07] | -1.91[-4.25; 0.5] | -1.85[-3.87; 0.2] | -2.03[-3.92; -0.09] | 3.88 [1.18; 6.65] | 1.48 [-1.83; 4.9] | 2.29 [0.32; 4.3] | 0.84 [-1.42; 3.15] | 2.25 [0.6; 3.93] | 1.57 [-0.28; 3.46] | 1.87 [-1.67; 5.53] | 2.7 [0.1; 5.37] | -3.08 [-4.66; -1.46] |
| Divinópolis | -0.29 [-0.65; 0.07] | 0.32 [-0.02; 0.66] | 0.06 [-0.21; 0.33] | 2.39 [0.49; 4.33] | 2.71 [1.75; 3.67] | 2.56 [1.63; 3.51] | 1.23 [0.32; 2.15] | 2.76 [0.86; 4.7] | 1.95 [0.64; 3.28] | -2.75[-4.26; -1.21] | -1.95[-3.32; -0.56] | -2.11[-2.93; -1.28] | 1.01 [-0.69; 2.74] | 1.39 [-1.57; 4.45] | 1.2 [-0.84; 3.29] | 1.43 [0.36; 2.51] | 0.08 [-0.94; 1.11] | 0.44 [-0.33; 1.23] | -0.29 [-1.19; 0.62] | -0.57 [-2.2; 1.08] | -2.23 [-3.96; -0.47] |
| Vitória* | -0.54 [-0.74; -0.33] | -0.64 [-0.83; -0.44] | -0.62 [-0.81; -0.43] | -0.12 [-1.77; 1.56] | -0.36 [-1.07; 0.34] | -0.35 [-1.03; 0.35] | 1.79 [0.46; 3.13] | 2.07 [1.34; 2.81] | 1.91 [1.14; 2.69] | -3.59[-4.48; -2.7] | -3.47[-4.22; -2.71] | -3.53[-4.16; -2.89] | 2.5 [0.97; 4.05] | 0.86 [-0.3; 2.04] | 1.44 [0.67; 2.21] | 0.63 [-0.5; 1.77] | -1.51 [-2.01; -1] | -0.99 [-1.49; -0.49] | 0.69 [-0.25; 1.64] | -0.81 [-1.62; 0.01] | -3.01 [-3.65; -2.37] |
| São Mateus | 0.96 [0.26; 1.68] | 1.7 [0.88; 2.53] | 1.4 [0.65; 2.15] | 2.14 [-0.03; 4.35] | 3.53 [1.42; 5.69] | 3.16 [1.17; 5.19] | 3.53 [1.36; 5.75] | 5.15 [2.53; 7.83] | 4.15 [2.15; 6.18] | -1.16[-2.75; 0.44] | -2.79[-3.69; -1.88] | -2.44[-3.18; -1.7] | 2.66 [-2.93; 8.57] | 7.47 [1.02; 14.34] | 4.38 [-0.4; 9.4] | 1.43 [-0.42; 3.31] | 0.35 [-1.27; 2] | 0.68 [-0.75; 2.13] | 3.95 [1.54; 6.41] | 3.83 [1.71; 6.01] | -1.2 [-3.41; 1.06] |
| Colatina | 0.22 [-0.67; 1.12] | 1.53 [0.5; 2.58] | 0.94 [0.19; 1.71] | 2.71 [-2.22; 7.88] | 2.81 [0.38; 5.3] | 2.46 [0.88; 4.06] | 1.03 [-1.19; 3.31] | 2.63 [1.01; 4.28] | 1.64 [0.3; 2.99] | -2.06[-4.03; -0.05] | -1.58[-3.35; 0.22] | -1.82[-3.53; -0.08] | -0.04 [-2.12; 2.08] | 1.94 [-3.57; 7.78] | 0.8 [-1.21; 2.85] | -0.17 [-1.8; 1.49] | 0.16 [-1.19; 1.54] | 0.05 [-1.26; 1.38] | 2.36 [-0.02; 4.81] | 3.61 [0.8; 6.5] | -1.59 [-2.76; -0.4] |
| Cachoeiro do Itapemirim | 0.24 [-0.88; 1.37] | 0.02 [-0.43; 0.47] | 0.11 [-0.44; 0.67] | -2.37 [-4.9; 0.23] | -0.35 [-1.55; 0.87] | -0.7 [-1.98; 0.59] | 3.2 [1.68; 4.74] | 2.85 [0.93; 4.81] | 2.95 [1.92; 3.98] | -1.54[-4.56; 1.57] | -3.34[-4.61; -2.06] | -2.85[-3.88; -1.81] | 3.18 [-0.79; 7.32] | 1.87 [0.27; 3.5] | 2.28 [0.97; 3.6] | 1.84 [0.3; 3.4] | -0.22 [-0.84; 0.4] | 0.38 [-0.25; 1.03] | 2.47 [0.46; 4.51] | 1.52 [0.08; 2.97] | -1.68 [-3.84; 0.53] |
| Rio de Janeiro* | -0.79 [-0.99; -0.59] | -1.29 [-1.54; -1.04] | -1.08 [-1.29; -0.87] | -1.74 [-2.45; -1.02] | -2.08 [-2.54; -1.61] | -2.08 [-2.59; -1.58] | 0.43 [-0.02; 0.88] | 0.84 [0.68; 1.01] | 0.67 [0.43; 0.9] | -2.76[-3.15; -2.37] | -3.12[-3.3; -2.93] | -3.01[-3.18; -2.83] | 1.17 [0.67; 1.67] | 0.37 [-0.2; 0.95] | 0.7 [0.23; 1.18] | 0.45 [0.18; 0.73] | -1.57 [-2.26; -0.88] | -1.96 [-2.16; -1.76] | -0.72 [-1.09; -0.36] | -0.82 [-1.56; -0.08] | -1.68 [-2.08; -1.29] |
| Volta Redonda - Barra Mansa | -0.34 [-0.62; -0.05] | -0.43 [-0.81; -0.06] | -0.4 [-0.65; -0.15] | 0.01 [-1.96; 2.01] | 0.29 [-0.62; 1.21] | 0.25 [-0.66; 1.17] | 0.99 [0.01; 1.98] | 3.11 [1.81; 4.43] | 2.02 [1.33; 2.73] | -3.05[-4.18; -1.91] | -2.37[-3.65; -1.07] | -2.55[-3.57; -1.51] | 1.22 [-0.38; 2.85] | 1.87 [-0.08; 3.85] | 1.32 [0.31; 2.34] | 1.25 [0.23; 2.28] | -1.42 [-2.13; -0.71] | -0.56 [-1.11; 0] | -0.05 [-0.64; 0.53] | -0.68 [-2.06; 0.72] | -1.3 [-2.95; 0.38] |
| Petrópolis | -0.74 [-1.1; -0.37] | -0.58 [-0.92; -0.24] | -0.66 [-0.94; -0.38] | -1.6 [-3.6; 0.43] | -0.19 [-0.9; 0.52] | -0.48 [-1.34; 0.38] | -0.01 [-0.96; 0.96] | 1.14 [0.35; 1.94] | 0.61 [-0.01; 1.23] | -2.69[-3.78; -1.59] | -2.78[-3.7; -1.84] | -2.74[-3.57; -1.9] | 1.03 [-0.62; 2.72] | 1.88 [0.55; 3.21] | 1.4 [0.47; 2.34] | 1.5 [0.9; 2.11] | -1.81 [-2.62; -0.99] | -0.79 [-1.38; -0.2] | 0.29 [-0.41; 1] | -0.15 [-0.71; 0.42] | -3.13 [-4.5; -1.75] |
| Campos dos Goytacazes | -0.54 [-0.77; -0.3] | -0.64 [-0.92; -0.36] | -0.6 [-0.79; -0.41] | -1.18 [-2.71; 0.38] | 0.15 [-1.02; 1.34] | -0.05 [-0.99; 0.91] | 1.1 [0.35; 1.87] | 1.56 [0.53; 2.6] | 1.32 [0.84; 1.8] | -1.31[-2.55; -0.05] | -2.72[-3.57; -1.86] | -2.32[-2.99; -1.65] | 1.07 [-0.04; 2.19] | -0.87 [-3.2; 1.52] | -0.08 [-1.69; 1.56] | -0.76 [-1.86; 0.36] | -0.9 [-1.7; -0.1] | -0.85 [-1.58; -0.11] | 0.35 [-0.32; 1.02] | 0.38 [-0.9; 1.67] | -2.64 [-3.5; -1.78] |
| Macaé - Rio das Ostras - Cabo Frio | -0.63 [-0.95; -0.31] | -0.62 [-0.94; -0.29] | -0.6 [-0.8; -0.4] | -2.99 [-6.48; 0.64] | -0.8 [-1.93; 0.34] | -1.23 [-2.67; 0.22] | 0.67 [-1.07; 2.44] | 2.33 [0.99; 3.7] | 1.45 [0.04; 2.88] | -3.69[-5.36; -1.98] | -2.46[-4.08; -0.8] | -3.07[-4.28; -1.84] | 2.53 [-0.82; 6] | 6.36 [-0.06; 13.19] | 2.07 [0.57; 3.59] | 1.53 [0.43; 2.63] | -0.77 [-1.84; 0.3] | -0.18 [-1.05; 0.7] | 1.05 [0.23; 1.87] | 0.21 [-0.28; 0.71] | -0.58 [-2.11; 0.98] |
| São Paulo* | -1.06 [-1.12; -1] | -1.46 [-1.52; -1.41] | -1.31 [-1.35; -1.26] | -1.06 [-1.65; -0.46] | -1.89 [-2.14; -1.64] | -1.78 [-2.02; -1.53] | -0.14 [-0.46; 0.18] | 0.47 [0.26; 0.69] | 0.2 [0.03; 0.37] | -2.78[-3.14; -2.42] | -3.44[-3.65; -3.23] | -3.25[-3.45; -3.05] | 0.36 [0; 0.73] | 0.28 [0.01; 0.55] | 0.3 [0.09; 0.52] | 0.96 [0.64; 1.27] | -2.14 [-2.41; -1.87] | -1.25 [-1.46; -1.03] | -1.28 [-1.41; -1.15] | -2.23 [-2.4; -2.05] | -3.43 [-3.99; -2.88] |
| Sorocaba | -0.97 [-1.18; -0.76] | -1.02 [-1.17; -0.87] | -1 [-1.11; -0.88] | -1.72 [-2.51; -0.93] | -0.96 [-1.42; -0.5] | -1.08 [-1.46; -0.71] | -0.5 [-1.27; 0.28] | 0.61 [0.22; 0.99] | 0.07 [-0.37; 0.51] | -4.54[-5.38; -3.7] | -4.38[-4.75; -4.02] | -4.43[-4.81; -4.05] | 0.66 [-0.37; 1.71] | 1.23 [0.06; 2.41] | 0.92 [0.03; 1.81] | 0.5 [-0.36; 1.36] | -1.8 [-2.12; -1.48] | -1.04 [-1.32; -0.76] | -0.93 [-1.6; -0.25] | -1.28 [-2.19; -0.35] | -3.95 [-4.99; -2.89] |
| Bauru | -0.43 [-0.68; -0.18] | -0.74 [-0.99; -0.49] | -0.59 [-0.83; -0.36] | -0.31 [-1.63; 1.04] | -1.46 [-2.16; -0.76] | -1.36 [-2; -0.71] | -0.08 [-0.85; 0.69] | 1.22 [0.47; 1.97] | 0.66 [0.02; 1.31] | -4.26[-5.33; -3.19] | -4.64[-5.76; -3.51] | -4.54[-5.43; -3.65] | 0.38 [-0.76; 1.52] | -0.28 [-1.54; 1.01] | -0.13 [-0.84; 0.58] | 1.88 [0.79; 2.97] | -1.47 [-2.52; -0.41] | -0.44 [-1.5; 0.63] | -1.24 [-2.2; -0.27] | -1.16 [-2.18; -0.12] | -4.6 [-5.85; -3.32] |
| Marília | -0.49 [-0.8; -0.17] | -0.63 [-1.02; -0.24] | -0.58 [-0.79; -0.36] | 1.48 [0.16; 2.82] | -1.13 [-2.3; 0.05] | -0.92 [-1.9; 0.07] | 0.24 [-0.66; 1.15] | 1.65 [0.34; 2.98] | 1.09 [0.02; 2.17] | -2.73[-4.42; -1] | -3.99[-5.45; -2.51] | -3.53[-5.03; -2.01] | -0.52 [-1.83; 0.8] | -0.25 [-1.55; 1.07] | -0.25 [-1.08; 0.59] | 1.13 [-0.17; 2.46] | -1.57 [-2.26; -0.88] | -0.71 [-1.4; -0.01] | -1.63 [-2.96; -0.28] | -1.47 [-2.83; -0.09] | -4.23 [-5.35; -3.09] |
| Presidente Prudente | -0.35 [-0.77; 0.06] | -0.2 [-0.49; 0.09] | -0.22 [-0.55; 0.11] | -0.13 [-1.87; 1.64] | -0.63 [-1.3; 0.04] | -0.56 [-1.25; 0.13] | 0.37 [-0.65; 1.4] | 0.98 [-0.18; 2.15] | 0.72 [-0.28; 1.74] | -3.1[-4.43; -1.76] | -3.87[-4.57; -3.16] | -3.63[-4.42; -2.84] | 0.27 [-1.54; 2.12] | 1.36 [0.03; 2.71] | 0.87 [0.01; 1.73] | 2.77 [1.87; 3.67] | -0.02 [-0.91; 0.88] | 0.72 [-0.02; 1.48] | -0.4 [-1.34; 0.56] | -0.43 [-1.29; 0.43] | -3.83 [-5.79; -1.82] |
| Araçatuba | -0.42 [-0.85; 0.01] | -0.89 [-1.33; -0.45] | -0.72 [-1.06; -0.37] | 0.23 [-1.53; 2.02] | -1.52 [-2.55; -0.48] | -1.35 [-2.28; -0.41] | -0.55 [-1.36; 0.28] | 1.42 [0.46; 2.39] | 0.51 [-0.11; 1.14] | -3.39[-4.69; -2.07] | -3.74[-4.9; -2.56] | -3.67[-4.45; -2.9] | -1.36 [-3.39; 0.71] | 1.02 [-0.05; 2.1] | 0.34 [-0.98; 1.68] | 1.65 [0.04; 3.29] | -1.32 [-1.92; -0.73] | -0.43 [-1.17; 0.31] | -0.64 [-1.57; 0.3] | -3 [-3.91; -2.08] | -3.89 [-6.83; -0.86] |
| São José do Rio Preto | -0.92 [-1.17; -0.67] | -0.83 [-1.11; -0.55] | -0.87 [-1.05; -0.68] | -1.13 [-2.01; -0.25] | -2.1 [-2.96; -1.23] | -2.02 [-2.71; -1.32] | -0.95 [-1.97; 0.08] | 1.2 [0.31; 2.1] | 0.23 [-0.5; 0.98] | -4.41[-5.7; -3.11] | -3.9[-5.36; -2.41] | -4.1[-4.9; -3.28] | -0.06 [-1.07; 0.96] | 0.81 [-0.66; 2.3] | 0.4 [-0.58; 1.39] | 1.14 [0.26; 2.03] | -1.17 [-1.75; -0.59] | -0.52 [-0.93; -0.1] | -1.21 [-1.77; -0.64] | -1.53 [-2.19; -0.88] | -4.08 [-5.28; -2.86] |
| Ribeirão Preto | -0.47 [-0.67; -0.27] | -0.82 [-1.13; -0.5] | -0.7 [-0.91; -0.49] | -0.51 [-1.48; 0.47] | -1.04 [-2.04; -0.02] | -1.02 [-1.92; -0.11] | 0.42 [-0.19; 1.04] | 1.54 [0.94; 2.14] | 1.05 [0.63; 1.48] | -3.34[-3.87; -2.81] | -3.88[-4.63; -3.12] | -3.74[-4.29; -3.2] | 1.41 [0.08; 2.77] | 0.91 [-0.13; 1.96] | 1.08 [0.49; 1.68] | 1.77 [1.11; 2.44] | -1.18 [-1.47; -0.88] | -0.37 [-0.64; -0.09] | -0.07 [-0.62; 0.48] | -1.04 [-1.8; -0.28] | -3.48 [-4.33; -2.62] |
| Araraquara | -0.1 [-0.48; 0.28] | -0.62 [-0.96; -0.27] | -0.4 [-0.6; -0.21] | -0.88 [-2.85; 1.13] | -1.14 [-1.6; -0.67] | -1.08 [-1.57; -0.6] | 0.27 [-0.92; 1.47] | 1.9 [0.57; 3.25] | 1.21 [0.31; 2.12] | -3.3[-5.01; -1.56] | -2.86[-3.73; -1.99] | -2.92[-3.73; -2.1] | 0.85 [-0.36; 2.06] | 0.44 [-1.03; 1.95] | 0.53 [-0.45; 1.51] | 2.54 [1.17; 3.93] | -0.81 [-1.53; -0.09] | 0.07 [-0.63; 0.78] | -0.9 [-1.7; -0.09] | -1.71 [-2.58; -0.84] | -2.93 [-4.01; -1.84] |
| Campinas | -0.81 [-0.97; -0.65] | -1.09 [-1.24; -0.94] | -0.98 [-1.12; -0.84] | -0.55 [-1.57; 0.48] | -1.72 [-2.2; -1.24] | -1.6 [-1.94; -1.26] | -0.24 [-0.65; 0.17] | 0.92 [0.44; 1.41] | 0.4 [0.06; 0.74] | -3.03[-3.48; -2.58] | -3.62[-4.14; -3.09] | -3.45[-3.93; -2.97] | 0.32 [-0.48; 1.12] | 0.1 [-0.41; 0.61] | 0.17 [-0.36; 0.7] | 0.74 [0.47; 1.02] | -1.86 [-2.29; -1.44] | -1.13 [-1.41; -0.85] | -1 [-1.58; -0.41] | -1.68 [-2.42; -0.93] | -3.66 [-4.39; -2.93] |
| São José dos Campos | -0.9 [-1.25; -0.54] | -0.82 [-1.08; -0.56] | -0.86 [-1.12; -0.6] | -1.77 [-3.02; -0.49] | -1.58 [-2.26; -0.89] | -1.63 [-2.17; -1.08] | 0.93 [-0.01; 1.88] | 1.24 [0.59; 1.89] | 1.15 [0.44; 1.87] | -4.02[-4.77; -3.27] | -3.69[-4.23; -3.15] | -3.8[-4.17; -3.43] | -0.09 [-1.25; 1.09] | 0.45 [-0.91; 1.82] | 0.23 [-0.71; 1.17] | 0.98 [0.29; 1.67] | -2.01 [-2.66; -1.36] | -1.1 [-1.65; -0.54] | -0.87 [-1.94; 0.23] | -0.06 [-0.85; 0.73] | -4.11 [-4.89; -3.31] |

* Intermediate regions containing state capitals ; Increasing trend, Decreasing trend, Stationary trend.

**Table S4: APC by intermediate region and cancer group, SOUTH region 1996-2016**

| **S** | **All Cancers** | | | **Head & Neck** | | | **Colon & Rectum** | | | **Stomach** | | | **Pancreas** | | | **Lung** | | | **Breast** | **Prostate** | **Cervix** |
| --- | --- | --- | --- | --- | --- | --- | --- | --- | --- | --- | --- | --- | --- | --- | --- | --- | --- | --- | --- | --- | --- |
|  | F | M | FM | F | M | FM | F | M | FM | F | M | FM | F | M | FM | F | M | FM | F | M | F |
| Curitiba* | -0.75 [-1.08; -0.42] | -0.82 [-1.23; -0.4] | -0.77 [-1.11; -0.42] | -2.17 [-2.94; -1.4] | -1.61 [-2.2; -1.02] | -1.7 [-2.21; -1.18] | 0.39 [-0.35; 1.14] | 0.81 [0.22; 1.41] | 0.65 [0.07; 1.23] | -3.1[-3.87; -2.31] | -3.01[-3.38; -2.63] | -3.03[-3.35; -2.7] | 1.04 [0.32; 1.75] | 0.33 [-0.4; 1.05] | 0.64 [0.16; 1.13] | 0.62 [0.1; 1.15] | -0.65 [-1.16; -0.14] | -0.24 [-0.61; 0.13] | -0.17 [-0.57; 0.24] | -0.62 [-1.57; 0.34] | -3.83 [-4.69; -2.96] |
| Guarapuava | 0.45 [-0.36; 1.27] | 0.51 [-0.54; 1.58] | 0.48 [-0.27; 1.24] | 1 [-3.49; 5.71] | 1.07 [-0.56; 2.73] | 0.87 [-0.5; 2.25] | 0.25 [-1.87; 2.41] | 1.12 [-0.75; 3.03] | 0.78 [-0.28; 1.86] | -3.69[-5.26; -2.09] | -1.27[-2.7; 0.19] | -2.07[-3.37; -0.75] | 2.81 [-0.32; 6.03] | 2.72 [-0.06; 5.57] | 2.6 [0.49; 4.76] | 3.78 [2.2; 5.39] | 0.49 [-1.06; 2.06] | 1.86 [0.88; 2.86] | 0.99 [0.06; 1.92] | 0.79 [-1.76; 3.4] | -1.11 [-3.07; 0.9] |
| Cascavel | 0.38 [0; 0.76] | -0.15 [-0.62; 0.34] | 0.07 [-0.3; 0.44] | -0.09 [-1.48; 1.33] | -0.4 [-1.39; 0.6] | -0.35 [-1.11; 0.42] | 2.3 [1.48; 3.12] | 3.35 [2.73; 3.98] | 2.82 [2.26; 3.39] | -2.04[-4.02; -0.01] | -2.57[-3.24; -1.88] | -2.55[-3.42; -1.68] | 2.06 [1; 3.14] | 1.09 [-0.12; 2.32] | 1.39 [0.75; 2.03] | 0.8 [-0.01; 1.62] | -0.91 [-1.84; 0.03] | -0.33 [-0.9; 0.25] | 1.66 [0.7; 2.62] | 0.35 [-0.52; 1.22] | -1.54 [-3; -0.06] |
| Maringá | -0.37 [-0.7; -0.05] | -0.3 [-0.58; -0.01] | -0.33 [-0.54; -0.12] | 0.05 [-1.63; 1.76] | -0.4 [-1.25; 0.46] | -0.38 [-1; 0.25] | 0.78 [-0.28; 1.85] | 1.55 [0.69; 2.41] | 1.21 [0.51; 1.92] | -3.97[-4.92; -3.01] | -3.32[-3.77; -2.86] | -3.52[-3.92; -3.13] | 0.55 [-0.52; 1.62] | 1.06 [-0.46; 2.6] | 0.83 [-0.04; 1.71] | 0.75 [0.24; 1.27] | -0.18 [-1.08; 0.73] | 0.12 [-0.59; 0.84] | 0.67 [0.23; 1.11] | -0.28 [-1.13; 0.57] | -2.49 [-3.18; -1.79] |
| Londrina | -0.16 [-0.35; 0.03] | -0.33 [-0.58; -0.09] | -0.27 [-0.43; -0.11] | -0.83 [-2.06; 0.41] | 0.07 [-0.59; 0.72] | -0.08 [-0.72; 0.56] | 1.04 [0.52; 1.56] | 1.33 [0.11; 2.56] | 1.23 [0.57; 1.89] | -3.29[-4.34; -2.22] | -3.37[-3.96; -2.78] | -3.34[-3.91; -2.77] | 2.29 [1.05; 3.54] | 1.17 [0.15; 2.21] | 1.62 [0.85; 2.41] | 0.59 [-0.2; 1.38] | -0.94 [-1.62; -0.25] | -0.49 [-0.96; -0.02] | 0.38 [-0.21; 0.96] | 0.25 [-0.16; 0.66] | -2.75 [-3.41; -2.08] |
| Ponta Grossa | -0.25 [-0.76; 0.27] | 0.38 [-0.06; 0.82] | 0.11 [-0.28; 0.5] | -2.77 [-4.52; -1] | -0.17 [-1.32; 1] | -0.74 [-1.91; 0.44] | 0.68 [-0.14; 1.5] | 2.12 [0.36; 3.92] | 1.45 [0.31; 2.6] | -2.01[-3.83; -0.16] | -1.35[-2.72; 0.04] | -1.57[-2.54; -0.59] | 0.96 [-0.19; 2.11] | 1.03 [-0.35; 2.43] | 0.93 [0.16; 1.7] | 0.78 [-0.32; 1.89] | -0.39 [-1.23; 0.46] | 0.13 [-0.67; 0.93] | 1.05 [0.05; 2.07] | 0.61 [-0.01; 1.24] | -1.38 [-3.69; 0.98] |
| Florianópolis* | -0.17 [-0.52; 0.18] | -0.55 [-0.83; -0.28] | -0.41 [-0.6; -0.22] | 0.88 [-2.17; 4.02] | -0.9 [-2.31; 0.54] | -0.72 [-1.9; 0.46] | -0.1 [-1.11; 0.93] | 0.93 [-0.04; 1.91] | 0.49 [-0.39; 1.38] | -0.11[-1.54; 1.35] | -3.49[-4.28; -2.7] | -2.46[-3.09; -1.82] | -0.01 [-1.08; 1.07] | 1.73 [-0.7; 4.22] | 0.86 [-0.44; 2.19] | 2.9 [1.43; 4.39] | -0.72 [-1.17; -0.27] | 0.07 [-0.3; 0.45] | -0.44 [-1.44; 0.57] | -1.74 [-2.49; -0.98] | -2.44 [-3.94; -0.92] |
| Criciúma | 0.03 [-0.43; 0.51] | -0.1 [-0.46; 0.26] | -0.07 [-0.34; 0.21] | 2.27 [-1.38; 6.06] | 0.74 [0.05; 1.42] | 0.94 [0.3; 1.58] | 0.46 [-0.89; 1.82] | 2.14 [0.79; 3.5] | 1.29 [0.62; 1.97] | -0.34[-1.92; 1.28] | -2.1[-3.53; -0.66] | -1.7[-2.98; -0.4] | 1.23 [-0.67; 3.17] | 0.52 [-0.61; 1.67] | 0.7 [-0.24; 1.65] | 2.38 [1.42; 3.34] | 0.62 [-0.51; 1.77] | 0.95 [0.21; 1.7] | 1.11 [-0.13; 2.36] | 0.11 [-1.52; 1.78] | -2.7 [-4.77; -0.59] |
| Lages | 0.41 [-0.1; 0.94] | 0.07 [-0.49; 0.62] | 0.2 [-0.11; 0.51] | -3.55 [-8.58; 1.75] | 0.73 [-2.45; 4.02] | -0.22 [-3.49; 3.16] | 0.99 [-1.27; 3.3] | 3.47 [1.28; 5.7] | 2.26 [0.95; 3.59] | -0.85[-2.59; 0.92] | -1.1[-2.43; 0.26] | -1.01[-2.07; 0.05] | 0.18 [-2.46; 2.88] | 1.72 [-3.22; 6.91] | -0.11 [-2.43; 2.26] | 2.21 [-0.44; 4.94] | -0.08 [-1.53; 1.39] | 0.74 [-0.37; 1.86] | 2.32 [1.13; 3.53] | -0.91 [-2.38; 0.58] | -0.97 [-3.78; 1.91] |
| Chapecó | -0.42 [-1.02; 0.18] | -0.98 [-1.35; -0.61] | -0.77 [-1.17; -0.37] | -1.33 [-3; 0.36] | -3.02 [-3.95; -2.08] | -2.77 [-3.62; -1.91] | 1.05 [-0.65; 2.78] | 2.04 [0.77; 3.33] | 1.54 [0.26; 2.83] | -4.17[-5.64; -2.68] | -3.78[-5.05; -2.49] | -3.89[-5.13; -2.64] | 0.76 [-1.24; 2.79] | -0.8 [-1.77; 0.17] | -0.13 [-1.14; 0.89] | 1.45 [0.41; 2.5] | -1.42 [-2.36; -0.47] | -0.73 [-1.56; 0.11] | 0.6 [-0.24; 1.44] | -0.86 [-2.58; 0.89] | -3.9 [-5.46; -2.31] |
| Caçador | -0.89 [-1.88; 0.12] | -1.15 [-1.95; -0.34] | -1.06 [-1.77; -0.34] | 1.49 [-2.08; 5.19] | -2.92 [-5.12; -0.67] | -2.14 [-4.24; 0] | 0.34 [-2.56; 3.32] | 2.75 [-2.8; 8.62] | 0.22 [-2.38; 2.89] | 0.76[-6; 8] | -3.14[-4.7; -1.54] | -2.93[-4.64; -1.19] | 0.83 [-3.74; 5.61] | -1.95 [-5.22; 1.42] | -0.99 [-3.74; 1.83] | 0.22 [-2.4; 2.92] | -1.28 [-2.66; 0.12] | -0.74 [-2.14; 0.69] | -1.27 [-3.61; 1.12] | -1.69 [-3.76; 0.42] | -1.61 [-5.09; 1.99] |
| Joinville | -0.12 [-0.7; 0.46] | -0.43 [-0.89; 0.03] | -0.31 [-0.78; 0.16] | -1.56 [-4.2; 1.14] | -1.05 [-2.16; 0.08] | -1.14 [-2.22; -0.04] | 0.35 [-0.98; 1.7] | 3.21 [1.85; 4.6] | 1.89 [0.61; 3.18] | -0.94[-2.37; 0.5] | -2.71[-3.41; -2] | -2.28[-2.93; -1.63] | 2.5 [0.36; 4.68] | 2.71 [1.11; 4.34] | 2.52 [0.97; 4.09] | 0.94 [-0.15; 2.05] | -0.79 [-1.75; 0.17] | -0.35 [-1.12; 0.42] | 0.58 [-0.42; 1.59] | -1.26 [-2.63; 0.12] | -1.16 [-3.39; 1.14] |
| Blumenau | -0.16 [-0.42; 0.11] | -0.42 [-0.78; -0.06] | -0.34 [-0.57; -0.11] | 1.73 [-0.9; 4.43] | -0.91 [-1.75; -0.06] | -0.63 [-1.5; 0.25] | 0.33 [-0.38; 1.04] | 1.27 [0.35; 2.2] | 0.8 [0.26; 1.34] | -3.63[-4.58; -2.68] | -3.76[-4.34; -3.17] | -3.73[-4.22; -3.24] | 0.58 [-2.08; 3.32] | 0.71 [-0.71; 2.15] | 0.63 [-0.62; 1.89] | 1.71 [0.58; 2.86] | -0.73 [-1.46; 0] | -0.14 [-0.87; 0.6] | 0.86 [0.18; 1.55] | 1.33 [-0.22; 2.9] | -2.13 [-2.92; -1.34] |
| Porto Alegre* | -0.71 [-0.93; -0.5] | -1.02 [-1.24; -0.8] | -0.9 [-1.1; -0.7] | -1.36 [-1.9; -0.82] | -1.47 [-1.89; -1.04] | -1.44 [-1.83; -1.05] | -0.13 [-0.46; 0.2] | 0.78 [0.36; 1.19] | 0.36 [0.02; 0.69] | -2.37[-3.07; -1.66] | -2.73[-3.31; -2.14] | -2.63[-3.06; -2.2] | 1.06 [0.19; 1.95] | 0.76 [0.1; 1.42] | 0.87 [0.15; 1.59] | 2.02 [1.34; 2.7] | -1.43 [-1.84; -1.02] | -0.5 [-0.93; -0.07] | -0.95 [-1.32; -0.58] | -1.26 [-1.63; -0.9] | -2.98 [-4.14; -1.81] |
| Pelotas | -0.41 [-0.81; -0.01] | -0.83 [-1.04; -0.63] | -0.68 [-0.91; -0.44] | -1.67 [-3.22; -0.1] | -0.32 [-0.9; 0.27] | -0.49 [-1.02; 0.04] | -0.25 [-0.86; 0.36] | 0.64 [-0.39; 1.68] | 0.2 [-0.5; 0.92] | -2.18[-3.41; -0.93] | -3.39[-4.37; -2.4] | -3.08[-3.76; -2.39] | 0.99 [-1.44; 3.47] | 1.15 [-0.5; 2.82] | 0.98 [-0.71; 2.69] | 1.7 [1.12; 2.28] | -2.21 [-2.6; -1.83] | -1.25 [-1.6; -0.9] | -0.04 [-0.53; 0.45] | -0.25 [-0.8; 0.29] | -2.18 [-3.24; -1.1] |
| Santa Maria | -0.45 [-0.74; -0.15] | -0.67 [-0.92; -0.42] | -0.59 [-0.83; -0.34] | -1.63 [-3.92; 0.71] | -1.6 [-2.8; -0.39] | -1.64 [-2.84; -0.43] | 0.08 [-1.2; 1.37] | 0.91 [0.02; 1.81] | 0.6 [-0.03; 1.25] | -3.96[-5.19; -2.71] | -2.2[-3.09; -1.31] | -2.71[-3.43; -1.99] | 0.9 [-0.61; 2.43] | 0.12 [-0.85; 1.09] | 0.53 [-0.47; 1.54] | 1.39 [0.76; 2.02] | -1.86 [-2.22; -1.49] | -0.95 [-1.27; -0.62] | -0.36 [-1; 0.29] | -0.72 [-1.75; 0.33] | -2.49 [-3.35; -1.62] |
| Uruguaiana | -0.76 [-0.95; -0.57] | -0.91 [-1.11; -0.71] | -0.86 [-1; -0.71] | 0.76 [-2.8; 4.44] | -0.33 [-1.83; 1.2] | -0.29 [-1.63; 1.07] | 1.18 [-0.05; 2.43] | 0.98 [-0.21; 2.2] | 1.11 [0.13; 2.09] | -2.84[-4.52; -1.13] | -3.18[-5.01; -1.32] | -3.13[-4.38; -1.87] | -0.38 [-2.4; 1.68] | 1.38 [-0.55; 3.34] | 0.53 [-0.95; 2.03] | 1.86 [1.04; 2.68] | -1.42 [-2.19; -0.64] | -0.41 [-1.07; 0.25] | -0.52 [-1.66; 0.64] | -0.33 [-1.88; 1.23] | -4.89 [-6.1; -3.66] |
| Ijuí | 0.16 [-0.19; 0.51] | -0.4 [-0.81; 0.01] | -0.18 [-0.48; 0.12] | -2.11 [-3.61; -0.6] | -2.39 [-3.62; -1.14] | -2.24 [-3.41; -1.06] | 1.36 [0.29; 2.44] | 2.26 [0.83; 3.72] | 1.67 [1.05; 2.28] | -3.23[-4.27; -2.18] | -2.74[-4.19; -1.27] | -2.92[-4.06; -1.77] | 0.24 [-1.15; 1.65] | 0.11 [-2.06; 2.32] | 0.07 [-1.35; 1.5] | 0.76 [-0.06; 1.59] | -1 [-1.71; -0.28] | -0.57 [-1.07; -0.06] | 0.95 [-0.25; 2.16] | -0.16 [-1.48; 1.18] | -0.78 [-3.11; 1.6] |
| Passo Fundo | -0.47 [-0.77; -0.17] | -0.62 [-1.22; -0.01] | -0.55 [-1.02; -0.08] | -1.57 [-3.31; 0.21] | -0.63 [-1.48; 0.23] | -0.76 [-1.6; 0.08] | 0.54 [-0.56; 1.66] | 1.34 [0.43; 2.27] | 1 [0.09; 1.92] | -1.88[-2.94; -0.81] | -2.44[-3.4; -1.47] | -2.2[-2.83; -1.57] | 0.13 [-1.22; 1.49] | 0.15 [-0.87; 1.19] | 0.16 [-0.6; 0.93] | 0.34 [-0.66; 1.36] | -1.17 [-1.71; -0.62] | -0.76 [-1.25; -0.27] | 0.96 [-0.02; 1.95] | -0.09 [-1.03; 0.86] | -2.33 [-3.28; -1.37] |
| Caxias do Sul | -0.51 [-0.96; -0.06] | -0.84 [-1.3; -0.37] | -0.72 [-1.06; -0.38] | -1.13 [-3.3; 1.08] | -1.82 [-2.37; -1.26] | -1.71 [-2.26; -1.15] | 0.06 [-0.56; 0.69] | 0.49 [-0.3; 1.27] | 0.31 [-0.06; 0.68] | -3.06[-4.17; -1.93] | -2.95[-3.58; -2.32] | -3.01[-3.46; -2.55] | 0.44 [-0.68; 1.58] | 1.03 [-1.04; 3.14] | 0.9 [-0.65; 2.48] | 2.19 [1.15; 3.25] | -1.53 [-2.44; -0.61] | -0.62 [-1.38; 0.16] | 0.14 [-0.74; 1.02] | -0.26 [-1.97; 1.47] | -3.08 [-4.32; -1.82] |
| Santa Cruz do Sul - Lajeado | -0.01 [-0.25; 0.23] | -0.2 [-0.52; 0.12] | -0.13 [-0.35; 0.09] | -0.95 [-2.34; 0.46] | -0.91 [-1.51; -0.3] | -0.91 [-1.52; -0.3] | 1.22 [-0.03; 2.48] | 1.5 [0.03; 3] | 1.39 [0.76; 2.03] | -3.15[-5.31; -0.95] | -3.94[-5.25; -2.61] | -3.76[-4.82; -2.68] | 2.73 [-0.61; 6.19] | 0.33 [-0.87; 1.54] | 0.84 [-0.71; 2.43] | 1.38 [0.01; 2.76] | -0.47 [-1.33; 0.4] | -0.06 [-0.94; 0.83] | 0.47 [-0.39; 1.33] | 0.27 [-0.75; 1.3] | -2.75 [-4.29; -1.18] |

* Intermediate regions containing state capitals ; Increasing trend, Decreasing trend, Stationary trend.

**Table S5: APC by intermediate region and cancer group, CENTER-WEST region 1996-2016**

| **CW** | **All Cancers** | | | **Head & Neck** | | | **Colon & Rectum** | | | **Stomach** | | | **Pancreas** | | | **Lung** | | | **Breast** | **Prostate** | **Cervix** |
| --- | --- | --- | --- | --- | --- | --- | --- | --- | --- | --- | --- | --- | --- | --- | --- | --- | --- | --- | --- | --- | --- |
|  | F | M | FM | F | M | FM | F | M | FM | F | M | FM | F | M | FM | F | M | FM | F | M | F |
| Campo Grande* | -1.01 [-1.32; -0.69] | -0.09 [-0.55; 0.36] | -0.47 [-0.77; -0.18] | -2.92 [-4.43; -1.39] | -0.26 [-1.21; 0.7] | -0.71 [-1.64; 0.22] | 0.81 [-0.09; 1.72] | 1.59 [0.77; 2.42] | 1.22 [0.47; 1.98] | -2.48[-3.55; -1.4] | -2.14[-2.76; -1.52] | -2.22[-2.85; -1.59] | 0.59 [-0.92; 2.13] | 2.6 [1.19; 4.03] | 1.72 [0.56; 2.9] | 0.05 [-1.25; 1.37] | -0.19 [-1.3; 0.94] | -0.17 [-0.92; 0.59] | 0.2 [-0.76; 1.16] | 0.26 [-1; 1.54] | -3.33 [-4.23; -2.42] |
| Dourados | -0.5 [-0.93; -0.07] | 0.49 [-0.26; 1.25] | 0.09 [-0.5; 0.69] | -1.78 [-3.86; 0.34] | 1.85 [0.01; 3.71] | 1.04 [-0.52; 2.61] | -0.66 [-2.83; 1.55] | 3.14 [0.91; 5.41] | 1.44 [-0.6; 3.52] | -4.13[-5.18; -3.07] | -2.67[-4.28; -1.03] | -3.18[-4.23; -2.13] | 2.23 [0.33; 4.17] | 0.94 [-1.86; 3.82] | 1.44 [-0.65; 3.57] | 1.18 [-0.52; 2.9] | 0.79 [-0.38; 1.99] | 0.91 [-0.11; 1.93] | 3.82 [1.12; 6.6] | 0.66 [0.05; 1.27] | -1.74 [-3.49; 0.04] |
| Corumbá | -0.13 [-1.04; 0.78] | 0.35 [-0.34; 1.04] | 0.09 [-0.53; 0.72] | -1.61 [-4.93; 1.82] | 2.61 [0.86; 4.39] | 1.76 [0.32; 3.23] | -0.3 [-1.74; 1.16] | -0.11 [-2.22; 2.04] | -0.04 [-1.2; 1.13] | 0.78[-2.52; 4.2] | -3.09[-5.7; -0.41] | -1.96[-3.87; -0.01] | -0.62 [-4.72; 3.66] | 2.63 [-0.69; 6.05] | 1.05 [-1.74; 3.92] | -1.79 [-5.02; 1.56] | 0.3 [-1.36; 1.98] | -0.39 [-2.02; 1.26] | 2.08 [0.44; 3.74] | 1.18 [0.13; 2.23] | 0.67 [-2.14; 3.56] |
| Cuiabá* | 0.12 [-0.39; 0.64] | 0.41 [-0.37; 1.19] | 0.28 [-0.33; 0.88] | 2.1 [-2.04; 6.42] | 1.49 [0.38; 2.61] | 1.42 [0.15; 2.71] | 2.79 [1.69; 3.91] | 2.5 [1.12; 3.89] | 2.56 [1.64; 3.48] | -1.52[-3.01; -0.01] | -2.39[-3.67; -1.08] | -2.22[-3.19; -1.24] | 2.96 [0.13; 5.87] | 1.67 [0.51; 2.84] | 2.25 [0.91; 3.62] | 1.1 [-0.03; 2.25] | -0.49 [-1.48; 0.5] | -0.01 [-0.55; 0.53] | 2 [0.32; 3.7] | 1.77 [0.14; 3.43] | -2.37 [-3.77; -0.94] |
| Cáceres | 0.3 [-0.61; 1.22] | 0.22 [-0.34; 0.78] | 0.23 [-0.23; 0.7] | 9.13 [-4.79; 25.08] | -0.61 [-4.18; 3.09] | -0.38 [-3.38; 2.7] | 5.19 [-0.4; 11.09] | 1.48 [-0.68; 3.7] | 2.05 [-0.5; 4.66] | -5.7[-11.5; 0.48] | -2.74[-4.22; -1.23] | -3.5[-4.94; -2.04] | 3.93 [-3.28; 11.67] | -0.14 [-3.71; 3.56] | 0.37 [-2.64; 3.48] | 3.95 [1.55; 6.41] | 0.12 [-2.55; 2.86] | 1.21 [-0.81; 3.27] | 1.7 [-2.19; 5.74] | 1.77 [-1.04; 4.66] | -1.57 [-5.08; 2.06] |
| Sinop | 1.25 [-0.01; 2.52] | 1.02 [-0.16; 2.22] | 1.21 [-0.16; 2.6] | 5.31 [-6.06; 18.05] | 3.51 [1.01; 6.08] | 3.03 [-0.19; 6.35] | 0.26 [-1.62; 2.18] | 2.73 [0.62; 4.88] | 1.44 [0.71; 2.18] | 0.79[-1.82; 3.46] | -4.56[-5.81; -3.29] | -3.27[-4.34; -2.19] | 11.66 [5.31; 18.39] | 8.48 [2.64; 14.65] | 8.64 [3.6; 13.92] | -0.47 [-1.8; 0.87] | 3.19 [-0.46; 6.97] | 1.91 [-0.44; 4.32] | 4.64 [2.59; 6.72] | -0.15 [-1.57; 1.28] | -2.6 [-4.54; -0.61] |
| Barra do Garças | -0.46 [-2.33; 1.44] | 0.79 [-0.24; 1.84] | 0.23 [-0.99; 1.45] | 9.67 [1.3; 18.72] | 1.28 [-1.93; 4.58] | 1.56 [-1.62; 4.85] | -0.06 [-3.36; 3.35] | 6.93 [-0.36; 14.75] | 2.43 [0.07; 4.85] | -2.32[-9.25; 5.14] | -5[-7.68; -2.24] | -4.32[-6.73; -1.84] | 2.03 [-3.48; 7.87] | 7.79 [-3.18; 19.99] | 3.88 [-1.9; 10] | 0.78 [-3.85; 5.64] | 0.52 [-3.08; 4.26] | -0.13 [-2.94; 2.76] | -1.65 [-4.52; 1.31] | 1.63 [-1.99; 5.38] | -6.48 [-9.33; -3.55] |
| Rondonópolis | 1.53 [0.22; 2.86] | 0.69 [-0.41; 1.81] | 0.94 [-0.08; 1.97] | 4.71 [-1.13; 10.9] | 3.71 [1.36; 6.11] | 3.42 [1.2; 5.69] | 5.2 [3.44; 6.98] | 3.33 [1.44; 5.25] | 4.06 [2.6; 5.53] | -2.81[-5.99; 0.49] | -2.32[-4.8; 0.21] | -2.7[-4.63; -0.74] | 1.77 [-2.19; 5.9] | -1.82 [-6.08; 2.62] | -0.46 [-2.88; 2.02] | 1.95 [-1.59; 5.61] | 0.36 [-2.65; 3.47] | 0.94 [-1.67; 3.63] | 7.37 [1.57; 13.5] | 0.95 [-0.9; 2.83] | -0.2 [-1.74; 1.36] |
| Goiânia* | 0.08 [-0.2; 0.36] | 0.47 [0.22; 0.71] | 0.3 [0.08; 0.53] | -0.6 [-1.88; 0.69] | 1.69 [0.93; 2.46] | 1.17 [0.48; 1.86] | 1.8 [1.04; 2.57] | 2.53 [1.95; 3.11] | 2.26 [1.77; 2.74] | -2.36[-3.34; -1.37] | -1.98[-2.37; -1.6] | -2.13[-2.46; -1.79] | 1.18 [-0.07; 2.45] | 2.22 [0.91; 3.55] | 1.75 [0.64; 2.87] | 1.24 [0.58; 1.9] | -0.09 [-0.57; 0.38] | 0.33 [-0.02; 0.69] | 1.64 [0.41; 2.89] | 0.76 [-0.31; 1.85] | -2.37 [-3.81; -0.91] |
| Itumbiara | 0.23 [-0.2; 0.67] | 0.34 [-0.39; 1.08] | 0.3 [-0.21; 0.81] | 1.25 [-0.95; 3.51] | 0.56 [-1.15; 2.3] | 0.56 [-0.67; 1.82] | 1.77 [-1.45; 5.1] | 4.38 [1.86; 6.97] | 2.87 [0.45; 5.35] | -1.6[-6.04; 3.04] | -4.1[-6.45; -1.69] | -3.74[-6.17; -1.25] | 6.15 [0.5; 12.12] | 0.51 [-3.57; 4.75] | 1.27 [-1.19; 3.78] | 0.6 [-2.06; 3.33] | -0.2 [-2.05; 1.69] | -0.05 [-1.38; 1.31] | 3.75 [1.73; 5.81] | 1.66 [0.11; 3.23] | -2.8 [-4.68; -0.89] |
| Rio Verde | -0.35 [-0.74; 0.04] | -0.09 [-0.44; 0.26] | -0.21 [-0.48; 0.06] | 0.63 [-2.62; 3.99] | 0.66 [-1.58; 2.94] | 0.42 [-1.36; 2.24] | 2.84 [1.37; 4.34] | 1.47 [-0.58; 3.57] | 2.18 [0.81; 3.56] | -0.19[-3.02; 2.72] | -2.92[-4.4; -1.42] | -2.16[-3.55; -0.74] | 0.79 [-2.34; 4.03] | 0.13 [-2.6; 2.93] | 0.15 [-1.41; 1.73] | 0.74 [-0.81; 2.31] | -0.1 [-0.89; 0.71] | 0.17 [-0.73; 1.08] | 0.68 [-1.26; 2.66] | 0.44 [-0.34; 1.23] | -2.71 [-4.27; -1.12] |
| São Luís de Montes Belos - Iporá | 0.46 [-0.27; 1.19] | 0.6 [-0.28; 1.49] | 0.48 [-0.19; 1.15] | 5.37 [-1; 12.15] | 4.1 [0.33; 8.01] | 2.97 [1.08; 4.9] | 2.13 [-0.09; 4.39] | 3.61 [0.74; 6.56] | 2.5 [1; 4.02] | -2.69[-6; 0.74] | -1[-3.5; 1.56] | -1.46[-3.55; 0.67] | 3.95 [-2.42; 10.73] | 4.83 [-1.87; 11.98] | 2.89 [0.52; 5.31] | 1.41 [-0.3; 3.15] | 1.2 [0.22; 2.19] | 1.16 [0.39; 1.93] | 3.35 [0.38; 6.4] | 0.79 [-0.84; 2.44] | -0.98 [-3.74; 1.85] |
| Porangatu - Uruaçu | 0.43 [-0.31; 1.18] | 1.18 [0.5; 1.87] | 0.85 [0.26; 1.44] | 1.66 [-2.15; 5.62] | 1.19 [-0.6; 3.02] | 1.15 [-0.61; 2.95] | 2.93 [0.92; 4.98] | 2.34 [-0.12; 4.86] | 2.42 [1.02; 3.84] | -2.45[-4.39; -0.48] | -0.05[-1.83; 1.76] | -1.05[-2.1; 0.01] | 2.98 [-2.26; 8.5] | 0.74 [-3.19; 4.84] | 1.45 [-2.38; 5.43] | 1.89 [-0.01; 3.82] | 0.32 [-1.32; 1.99] | 0.91 [-0.21; 2.04] | 4.33 [1.26; 7.48] | 1.36 [0.11; 2.63] | 0.11 [-1.44; 1.68] |
| Luziânia - Águas Lindas de Goiás | 1.04 [0.36; 1.73] | 0.8 [0.15; 1.45] | 0.92 [0.38; 1.46] | -0.29 [-2.59; 2.08] | 1.95 [0.06; 3.87] | 1.36 [-0.57; 3.33] | 3.21 [0.95; 5.53] | 0.81 [-0.28; 1.9] | 1.71 [0.71; 2.73] | 1.48[-0.74; 3.75] | -1.63[-2.99; -0.26] | -0.91[-2; 0.19] | 4.71 [-1.64; 11.46] | 5.09 [2.1; 8.17] | 4.37 [0.76; 8.11] | 3.41 [1.45; 5.41] | 0.61 [-0.57; 1.81] | 1.5 [0.37; 2.63] | 2.27 [-0.05; 4.64] | 1.3 [-0.32; 2.96] | -0.96 [-2.42; 0.53] |
| Distrito Federal | -1.25 [-1.62; -0.87] | -1.61 [-1.92; -1.3] | -1.46 [-1.73; -1.18] | -0.26 [-1.5; 0.99] | -1.22 [-1.94; -0.5] | -1.03 [-1.77; -0.28] | 0.24 [-0.92; 1.42] | 1.19 [0.44; 1.94] | 0.73 [0.05; 1.41] | -1.62[-2.74; -0.49] | -2.75[-3.65; -1.85] | -2.31[-3.07; -1.54] | 0.03 [-1.82; 1.9] | -0.56 [-1.66; 0.56] | -0.36 [-1.54; 0.83] | -0.35 [-0.98; 0.29] | -2.12 [-2.75; -1.48] | -1.55 [-2.02; -1.08] | -0.58 [-1.09; -0.06] | -2.33 [-3.12; -1.53] | -2.83 [-3.59; -2.07] |

* Intermediate regions containing state capitals ; Increasing trend, Decreasing trend, Stationary trend.

**Table S6**. Pearson correlation of Human Development Index and APC by cancer type and macro-region

|  | **Sex** | **North**  **(n.22)** | **Northeast**  **(n.42)** | **Southeast**  **(n.33)** | **South**  **(n.21)** | **Center-West**  **(n.15)** |
| --- | --- | --- | --- | --- | --- | --- |
| **All cancers** | **F** | -0.80** | -0.75** | -0.86** | -0.08 | -0.45 |
|  | **M** | -0.69** | -0.77** | -0.87** | -0.22 | -0.73* |
| **Head & Neck** | **F** | 0.21 | -0.21 | -0.56** | 0.24 | 0.04 |
|  | **M** | -0.10 | -0.49** | -0.84** | -0.30 | -0.44 |
| **Colon, Rectum & Anus** | **F** | 0.40 | -0.22 | -0.77** | -0.25 | -0.04 |
|  | **M** | 0.63* | -0.36* | -0.74** | 0.08 | -0.27 |
| **Stomach** | **F** | -0.62* | -0.39* | -0.77** | 0.37 | 0.02 |
|  | **M** | -0.54* | -0.48** | -0.86** | -0.34 | 0.08 |
| **Pancreas** | **F** | 0.31 | -0.31* | -0.36 | -0.19 | -0.34 |
|  | **M** | 0.34 | -0.29 | -0.24 | 0.05 | -0.30 |
| **Lung** | **F** | -0.20 | -0.30 | -0.54** | 0.02 | -0.16 |
|  | **M** | -0.50* | -0.75** | -0.84** | -0.04 | -0.72* |
| **Breast** | **F** | -0.02 | -0.60** | -0.84** | -0.11 | -0.03 |
| **Prostate** | **M** | -0.29 | -0.55** | -0.64** | -0.09 | -0.17 |
| **Cervical** | **F** | -0.44 | -0.66** | -0.87** | -0.24 | -0.69* |

(*) P for trend < 0.05; (**) P for trend < 0.001
